# Integrating indoor and outdoor nitrogen dioxide exposures in US homes nationally by ZIP code

**DOI:** 10.1093/pnasnexus/pgaf341

**Published:** 2025-12-02

**Authors:** Yannai Kashtan, Chenghao Wang, Kari C Nadeau, Robert B Jackson

**Affiliations:** Earth System Science Department, Stanford University, 473 Via Ortega, Stanford, CA 94305, USA; PSE Healthy Energy, 1440 Broadway, Suite 750, Oakland, CA 94612, USA; School of Meteorology, University of Oklahoma, 120 David L. Boren Blvd, Norman, OK 73072, USA; Department of Geography and Environmental Sustainability, University of Oklahoma, 100 E. Boyd Street, Norman, OK 73019, USA; T.H. Chan School of Public Health, Harvard University, 677 Huntington Ave, Boston, MA 02115, USA; Earth System Science Department, Stanford University, 473 Via Ortega, Stanford, CA 94305, USA; Woods Institute for the Environment and Precourt Institute for Energy, Stanford, CA 94305, USA

**Keywords:** indoor air, natural gas, pollution, exposure, stove

## Abstract

Concentrations of nitrogen dioxide (NO_2_) measured outdoors using satellites and ground-level stations across the United States are regularly used to estimate NO_2_ exposures and disease burdens. In contrast, exposures attributable to sources of NO_2_ indoors are neither systematically monitored nor estimated. Here, to our knowledge we produce the first nationwide, ZIP-code-level estimate of total residential NO_2_ exposure that integrates both outdoor sources and the primary indoor source—gas- or propane-burning stoves. To estimate exposure by ZIP code, we combine our measurements of indoor NO_2_ emissions and concentrations in more than 15 cities across seven regions with outdoor NO_2_ concentrations and comprehensive housing stock data for 133 million residential dwellings and statistical samplings of occupant behavior. We estimate average total residential long-term NO_2_ exposure across the United States to be ∼10 ppbv for people who own a gas stove (∼18 ppbv or more for households in the top 5% of gas burned while cooking) and ∼8 ppbv from total outdoor exposure for those with electric stoves, which cause no additional NO_2_ exposure. Across the United States, the NO_2_ exposure of ∼22 million people would fall below the World Health Organization (WHO)'s long-term exposure guideline (10 µg/m^3^ or 5.2 ppbv) if they reduced or stopped cooking with gas or propane. Gas and propane stoves are also responsible for virtually all (>99%) of the residential exceedances of the WHO's 1-h-averaged air quality guideline across the United States. Gas and propane stoves are a substantial source of residential NO_2_ exposure even when compared with all outdoor sources combined.

Significance Statement Here, we show that stoves account for one quarter of total long-term indoor and outdoor exposure of nitrogen dioxide—an asthma trigger—for people who cook with gas or propane in the United States. Their indoor exposure can jump to more than half of their total if they cook more often or more intensely than average. We also find that gas stoves are responsible for virtually all cases when people's exposure at home surpasses 1-h high-intensity “acute” health guidelines. Our ZIP-code-level maps of exposure should help identify regions to prioritize for indoor and outdoor air quality interventions, particularly in lower-income communities.

## Introduction

Long-term exposure to nitrogen dioxide (NO_2_) has been linked to increased incidence and exacerbation of childhood asthma (1–4), incidence and mortality from long-term obstructive pulmonary disease (COPD) (5–7), and incidences of lung cancer, preterm birth, and diabetes mellitus (8). Primary outdoor sources of NO_2_ in the United States include road traffic exhaust, combustion of fossil fuels for electricity generation, and natural gas flaring. Indoors, NO_2_ is generated primarily from the combustion of natural gas or propane in stoves and other gas appliances (9–12).

Location-specific estimates of NO_2_ exposure outdoors modeled from actual NO_2_ concentrations are widely available (2, 3, 13). Such estimates cover much of the globe and have been used to calculate regional pediatric asthma burdens and to identify geographic regions of highest concern (2, 3, 13). However, such tools are of limited use for estimating total NO_2_ exposure and resulting disease burdens because they omit indoor sources, particularly emissions from gas stoves, and because they assume that NO_2_ or other outdoor pollutants would infiltrate indoor spaces independent of local climate and house characteristics. Indeed, outdoor NO_2_ concentrations alone have been poor predictors of full NO_2_ exposure (14–17).

Despite estimates of indoor NO_2_ concentrations and exposure found in the literature, we know of no US or global indoor maps equivalent to the existing maps of outdoor NO_2_ concentration and thus no means to compare indoor and outdoor NO_2_ exposures systematically or to combine them to obtain total exposure nationally or regionally. Previous studies have quantified the emission rates of NO_2_ and other pollutants from gas and propane stoves (9–11) and have measured indoor and outdoor NO_2_ concentrations in various dwelling types (10, 18–28). Previous empirical studies have also quantified the ratio of indoor to outdoor NO_2_ for individual US and international cities (17, 29), measured personal exposure in different cities (30–32), and modeled indoor and outdoor exposure in specific geographies but not nationally (30, 33–35).

As outdoor NO_2_ concentrations in the United States continue to fall because of stronger pollution controls (36), the relative burden of NO_2_ exposure attributable to indoor sources is likely to climb, as will the discrepancy between exposure inferred from outdoor concentrations alone and actual exposure. We previously constructed a model of stove-attributable NO_2_ exposure (12) at the state level but did not estimate exposures nationally or compare exposures indoors to those from outdoor sources. Here, to address the gap between outdoor concentrations alone and actual exposure, we adapted our model to estimate stove-attributable exposure at the ZIP-code level and to account for infiltration of outdoor NO_2_. We combined our indoor exposure estimates with ZIP-code-specific datasets of outdoor NO_2_ concentrations, housing stock, and climate to produce US-wide maps of estimated long- and short-term total residential NO_2_ exposure (NO_2_ exposure excluding those experienced in vehicles or at work). Our new exposure estimates provide a more complete picture of NO_2_ exposures than maps of outdoor NO_2_ concentrations alone and may help decision-makers prioritize locations for building electrification and ventilation retrofits. Our methodology may also be extended in the future to estimate exposures to other gas stove pollutants including benzene, carbon monoxide (CO), formaldehyde, and ultrafine particulate matter (37–40).

## Results

### Total long-term NO_2_ exposure attributable to stoves and to outdoor sources across US ZIP codes

**Fig. 1.**
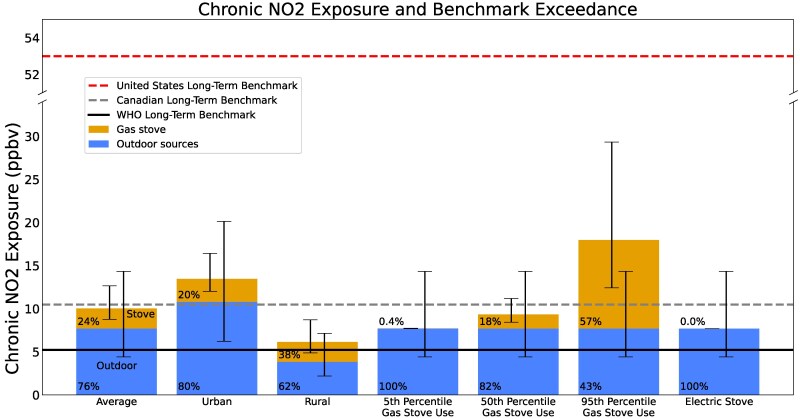
Indoor (gold; top) NO_2_ and outdoor (blue; bottom) average NO_2_ exposures. Long-term residential NO_2_ exposure attributable to outdoor sources (bottom portion of each bar) and combustion by gas and propane stoves (top portion of bar) across the US population with gas or propane stoves. Percentages indicate the percentage of total residential long term NO_2_ exposure due gas stoves (when over top portion of bar) and outdoor sources (when bottom blue portion of bar). From left to right: “Average” averaged across all US ZIP codes, “Urban” for all people in urban US ZIP codes (defined as being in the top 10% of US population density), “Rural” for all people in rural US ZIP codes (defined as being in the bottom 10% of US population density); “5th percentile,” “50th percentile,” and “95th percentile” for households with gas stoves in the 5th, 50th, and 95th percentile of gas stove use intensity (see Supplementary Definitions), respectively, across all US ZIP codes; “electric stove” for households with electric stoves. The black horizontal solid line represents the WHO long-term NO_2_ exposure guideline (10 µg/m^3^ or 5.2 ppbv), the gray dashed line represents the Canadian long-term NO_2_ exposure guideline (20 µg/m^3^ or 11 ppbv) (41), and the red dashed line represents the US EPA's long-term NO_2_ concentration standard (53 ppbv) (42). Error bars represent 95% CIs (see Materials and methods).

We find that gas and propane combustion in stoves is a substantial source of NO_2_ exposure indoors relative to outdoor sources. We estimate the population-averaged residential NO_2_ exposure from gas and propane stoves to be 2.4 (95% CI: 1.1–5.0) ppbv and the total population-averaged residential NO_2_ exposure (from gas and propane stoves and outdoor sources combined) to be 10.1 (95% CI: 5.5–14.6) ppbv among households with gas or propane stoves. Gas and propane stoves thus represent a quarter of total average residential NO_2_ exposure for people who use gas stoves. Outdoor sources are responsible for 7.7 (95% CI: 4.4–14.4) ppbv of NO_2_ exposure on average nationally, the remaining three quarters (see Fig. 1 and Table 1).

**Table 1.** Summary of modeled long-term NO_2_ exposure (in ppbv) in the United States for people with gas or propane stoves.

	Stove-attributable, population average	Stove-attributable, 5th percentile use	Stove-attributable, 50th percentile use	Stove-attributable, 95th percentile use	Outdoor attributable
Mean	2.4 (95% CI: 1.1–5.0)	0.03 (95% CI: 0.01–0.07)	1.7 (95% CI: 0.8–3.5)	10.3 (95% CI: 4.8–21.7)	7.7 (95% CI: 4.4–14.3)
Median	2.3 (95% CI: 1.1–4.8)	0.0 (95% CI: 0.0–0.1)	1.6 (95% CI: 0.7–3.3)	10.1 (95% CI: 4.7–21.3)	4.9 (95% CI: 2.8–9.2)
Max	4.2 (95% CI: 1.9–8.8)	0.0 (95% CI: 0.0–0.1)	3.0 (95% CI: 1.4–6.3)	17.5 (95% CI: 8.1–36.9)	17.9 (95% CI: 10.3–33.5)
Min	1.7 (95% CI: 0.8–3.6)	0.0 (95% CI: 0.0–0.1)	1.2 (95% CI: 0.6–2.6)	6.9 (95% CI: 3.2–14.6)	0.3 (95% CI: 0.2–0.6)

“Mean” indicates the population-weighted average across all ZIP codes; “median” corresponds to the ZIP code with the median population-averaged value among all ZIP codes; “max” and “min” correspond to the ZIP codes with the maximum and minimum population-averaged values, respectively. Columns represent population-averaged values (leftmost) and values corresponding with the 5th, 50th, and 95th percentiles of gas stove use intensity (see Supplementary Definitions), and for outdoor-attributable exposure only. For instance, the cell in the “5th percentile” column and “Max” row shows the average exposure among households in the 5th percentile of gas stove use in the ZIP code with the greatest stove-attributable exposure. Outdoor-attributable exposure is identical to exposure for people with electric stoves because electric stoves emit no NO_2_. The range in brackets is the 95% CI.

**Fig. 2.**
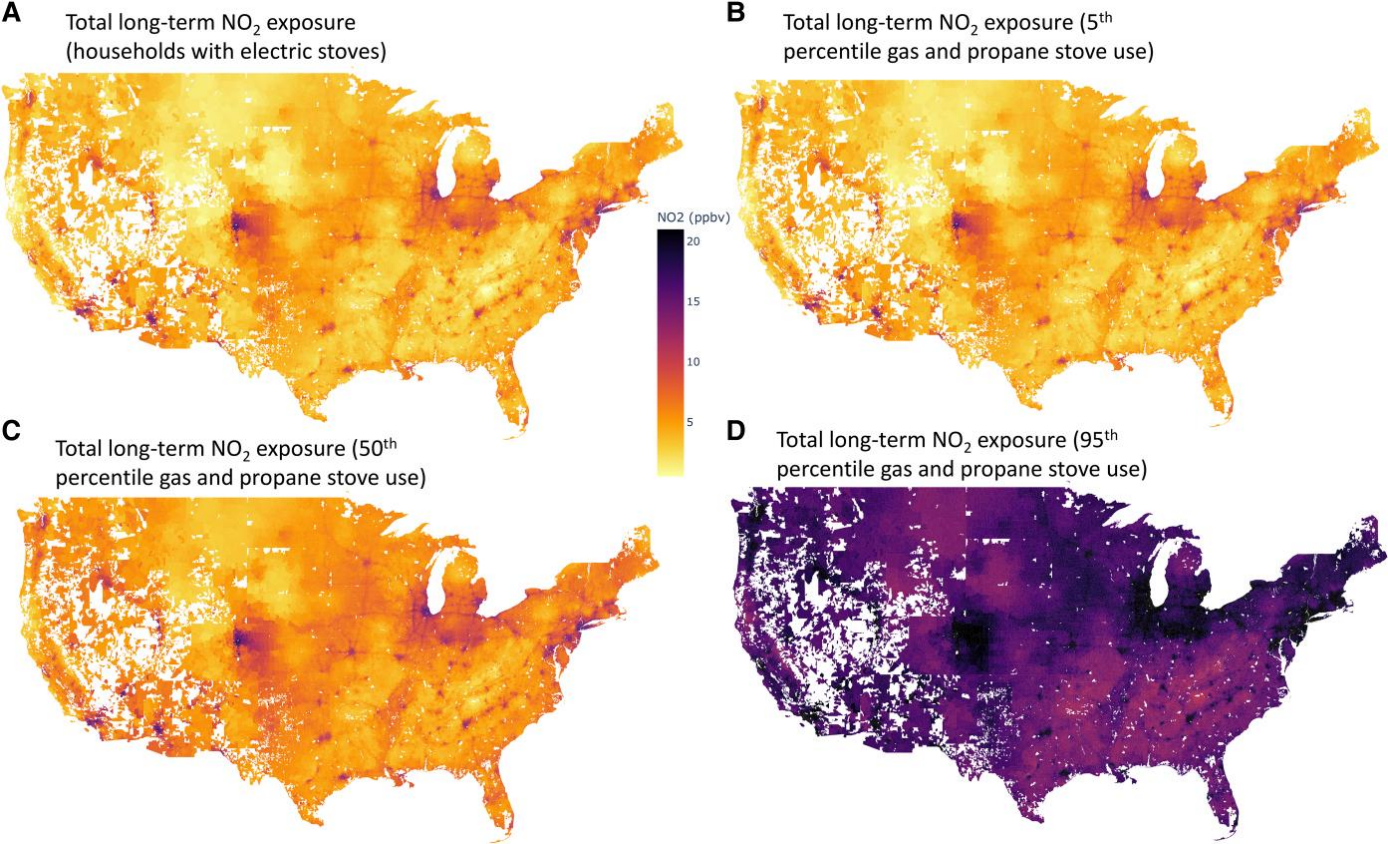
ZIP-code level estimates of total residential NO_2_ exposure in ppbv (indoor and outdoor) for (A) households who cook exclusively with an electric stove (exposure from outdoor sources only); (B) 5th, (C) 50th, and (D) 95th percentiles of fuel use for households with gas or propane stoves who cook in the 5th, 50th, and 95th percentiles of fuel use (see Supplementary Definitions). Gaps in white represent locations that do not have ZIP codes.

Indoor NO_2_ exposure from gas and propane stoves depends in part on how much gas is burned while cooking and how much time people spend in the kitchen. Stratifying exposure by gas stove use intensity (i.e. by amount of gas burned while cooking, see Supplementary Definitions), we find that people in households burning the 5th, 50th, and 95th percentiles of fuel nationally on average are exposed to 0.03 (95% CI: 0.01–0.07) ppbv, 1.7 (95% CI: 0.8–3.5) ppbv, and 10.3 (95% CI: 4.8–21.7) ppbv, respectively, of additional NO_2_ exposure from using their gas or propane stoves (Fig. 2). For 95th percentile households, gas stoves are responsible for more than half (57%) of total NO_2_ exposure combined indoors and outdoors. In contrast, electric stoves emit no NO_2_ and therefore contribute no additional NO_2_ exposure (12).

How long people spend in the kitchen also affects exposure. However, because pollution spreads quickly throughout homes, time spent in the kitchen impacts exposure less than how much gas is burned or a household's ventilation habits (12). People in the 95th percentile of time spent in the kitchen (2.5 h/day whether or not they are actively cooking, see Supplementary Definitions) are exposed to roughly 25% more long-term stove-attributable NO_2_ than people who spend a median amount of time in the kitchen (35 min/day), all else held constant, and roughly 31% more long-term NO_2_ than people in the 5th percentile of time spent in the kitchen (5 min/day) (43).

Gas and propane stoves increase exposure more in some ZIP codes than in others depending on housing characteristics and climate (Fig. S4). Among households with gas or propane stoves, we estimate that population-weighted average long-term NO_2_ exposure from gas stoves varies from a low of 1.69 ppbv to a high of 4.16 ppbv across US ZIP codes, a range driven by a combination of average residence size, air exchange with the outdoors, and climate. Modeled NO_2_ exposure attributable to gas stove combustion is modestly but significantly related to NO_2_ exposure from outdoor sources (*P* < 0.001; *r*^2^ = 0.14, see Fig. S2 for regression statistics). This relationship may be because more densely populated ZIP codes have both denser apartment-style housing and smaller rooms (44) as well as higher outdoor NO_2_ concentrations from urban sources (45) (Figs. S5−S18, Table S3). Indeed, population density is correlated with higher modeled NO_2_ exposures due to both outdoor sources (*r*^2^ = 0.21, *P* < 0.001) and gas and propane stoves (*r*^2^ = 0.33, *P* < 0.001).

### Exceedance of long-term NO_2_ exposure guidelines from the World Health Organization and Environmental Protection Agency

**Fig. 3.**
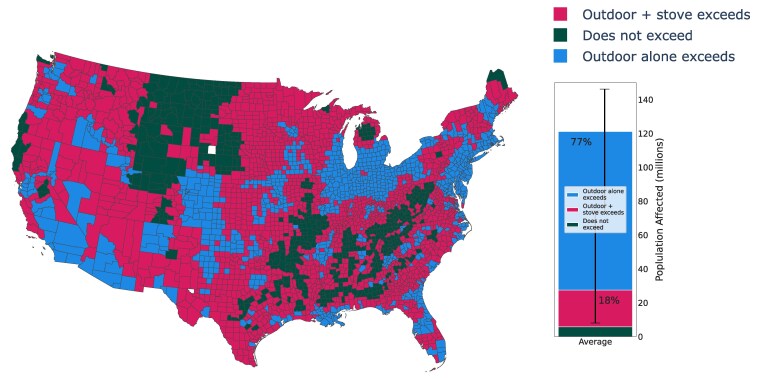
Stove-attributable exceedance of long-term NO_2_ benchmarks by county and population. US counties (map, left) and US population (bar, right) that exceed the WHO long-term NO_2_ exposure guideline: blue indicates counties where the average outdoor-attributable residential exposure alone is above the WHO long-term guideline, regardless of indoor pollution sources; magenta indicates counties where outdoor attributable residential NO_2_ exposure alone falls below the exposure guideline but the addition of exposure from gas stoves increases total residential exposure above the WHO guideline; dark green indicates counties where the sum of average outdoor- and stove-attributable exposure remains below the WHO guideline. Error bars on the right represent 95% CIs (see Materials and methods).

Approximately 77% of the US population is exposed to enough NO_2_ from outdoor sources alone to exceed the World Health Organization (WHO)'s long-term exposure guideline of 10 µg/m^3^ (5.2 ppbv), which applies to “both outdoor and indoor environments globally” (46) (see Fig. 3). Of the ∼27 million people in the United States whose NO_2_ exposure from outdoor sources alone falls below the WHO's guideline but who own a gas stove, using their stove increases their total residential exposure above the WHO's guideline for 22 (95% CI: 2.3–69) million people nationally, or 79% of such cases. In other words, we estimate that the long-term NO_2_ exposure of ∼22 million people nationally would fall below the WHO's long-term exposure guideline only by switching from gas to electric cooking. The area affected nationally is disproportionately large: in just under half (42%) of US ZIP codes and more than half (54%) of US counties, on average, the combination of NO_2_ exposure from outdoor sources and from gas or propane stoves causes total long-term residential NO_2_ exposure to exceed the WHO's long-term guideline (Fig. 3).

The US Environmental Protection Agency (EPA)'s long-term concentration standard (53 ppbv) applies outdoors and is roughly ten times higher than the WHO's exposure guideline, which applies both indoors and outdoors (42). While the majority of gas stove owners' exposure stays below the EPA standard, individuals living in <1,000 sq. ft. apartments whose gas stove use is in the top 5% receive at least 33 ppbv of long-term NO_2_ exposure from their gas stove. This stove-attributable NO_2_ exposure is sufficient to raise this population's total NO_2_ exposure close to the EPA's standard in the most polluted ten percent of ZIP codes, where outdoor-attributable NO_2_ exposure accounts for between 10 and 18 ppbv of additional exposure.

### Exceedance of the 1-h NO_2_ exposure guidelines from the WHO and EPA

In the Uninted States, outdoor sources alone cause virtually no exceedances of the WHO's hour-averaged NO_2_ exposure threshold of 200 µg/m^3^ (∼100 ppbv) (46). Between 2019 and 2022, the US EPA's nationwide network of over 450 real-time NO_2_ monitors concentrated in high-pollution areas detected hour-averaged outdoor NO_2_ concentrations exceeding 100 ppbv in fewer than one in 100,000 of all measurements, with all exceedances concentrated in seven monitors (47) (Fig. S3).

People who live in homes with a gas stove, whether or not they are the primary cook, are exposed to hour-averaged NO_2_ concentrations exceeding 100 ppbv on 7–15 days per year on average from the gas stove alone (see Materials and methods; these exceedances are exclusively due to 95th percentile cooking weeks). Meanwhile, occupants who are present exclusively during weeks with gas stove use intensity in the 95th percentile (see Supplementary Definitions) are exposed to hour-averaged concentrations over 100 ppbv in more half of prevalence-weighted modeled scenarios (i.e. more than half the time, see Fig. S19). Occupants present during weeks in the 5th and 50th percentiles of gas stove use intensity in contrast are never exposed to estimated hour-averaged concentrations exceeding 100 ppbv. The US EPA's outdoor short-term concentration standard is defined as no more than 2% of days with a maximum hour-averaged NO_2_ concentration over 100 ppbv over a 3-year period (7 days per year on average) (42). Thus, if the EPA's standards were used as a benchmark for indoor exposure, gas and propane stoves would be responsible for virtually all the exceedances of the EPA short-term benchmark. Moreover, households in the 95th percentile of stove use intensity would exceed the EPA's 100 ppbv benchmark most days of the year on average.

### Literature context

We estimated mean outdoor-attributable NO_2_ exposures to be 7.7 ppbv and total NO_2_ exposures to be 8.6 ppbv across the United States, including people with nonemitting electric stoves, for an outdoor/total ratio of 0.89. Among people with gas and propane stoves, total NO_2_ exposure is 10.1 ppbv. A recent review of the empirical literature from 2000 to 2016 conducted by Evangelopoulos et al. (17) on 10 North American studies found mean outdoor-attributable and total NO_2_ exposures among people with gas, propane, and electric stoves to be 12.0 and 16.7 ppbv, respectively, for an outdoor/total ratio of 0.74. Our lower estimates of outdoor-attributable NO_2_, 7.7 ppbv compared with 12.0 ppbv, are likely due to falling outdoor NO_2_ concentrations, which dropped from ∼16 ppbv on average in 2000 to ∼8 ppbv in 2016 (48).

Furthermore, Evangelopoulos et al.'s previous estimate of indoor NO_2_ exposure of 4.3 ppbv is nearly twice ours, likely because these older studies included additional sources of indoor NO_2_ such as unvented space heaters and because pilot lights were more common. We observed only one stove with a pilot light in our field work and thus did not model pilot lights. Pilot lights can raise average indoor NO_2_ concentrations by twice as much as gas stoves without pilot lights (26). Unvented gas heaters are subject to increasingly strict emission standards (49), meaning that they likely contribute to a smaller share of NO_2_ exposure today than in previous years. A decline in pilot lights and a drop in pollution from heaters both represent genuine decreases in exposure. Any remaining exposure attributable to ventless gas space heaters today is not captured by our model because we lack information on the geographical distribution of ventless space heaters. The true NO_2_ exposure among people who use unvented gas heaters is therefore higher than we estimate.

## Discussion

Breathing NO_2_ has been linked to childhood asthma, COPD, lung cancer, preterm birth, diabetes mellitus, and other health harms (1, 3, 5–8, 13). Although outdoor concentrations of NO_2_ are regularly monitored nationwide (47), corresponding indoor concentrations are neither systematically monitored nor estimated (2, 3, 13, 50), leading to underestimates of the health effects of breathing NO_2_ pollution from burning fossil fuels.

Our results show that gas and propane stoves in the United States are responsible for a substantial proportion of long-term NO_2_ exposure. When averaged across the entire US population (including the 60% of households that own electric stoves), gas and propane stoves are still responsible for an estimated 10% of total NO_2_ exposure across the United States. For households that own gas stoves, we estimate that gas and propane stoves contribute roughly one quarter of long-term NO_2_ exposure, consistent with the results from an earlier study undertaken solely in southern California (51). Moreover, we find that combustion by gas or propane stoves contributes roughly half or more of all long-term NO_2_ exposure for the ∼2.5 million households (52) in the top 5% of gas burned while cooking (see Supplementary Definitions).

We also estimate that gas and propane stoves in the United States are responsible for virtually all estimated short-term NO_2_ exposures that exceed the WHO's short-term guideline (200 µg/m^3^, or ∼100 ppbv, applied indoors and outdoors) and the US EPA's outdoor short-term concentration standard (100 ppbv) if it were applied indoors. This is primarily because NO_2_ exposure from gas stoves generally occurs as relatively brief exposures to high concentrations. We find that typical gas stove use would be sufficient to exceed the WHO's exposure guideline and the EPA's concentration standard (if applied indoors as an exposure benchmark) on 7–15 days/year on average. Indeed, gas stoves are responsible for virtually all residential exceedances of these guidelines. Because electric stoves emit no NO_2_, people in households with electric coil and induction stoves experience none of this additional exposure associated with burning fossil fuels indoors (12).

Additional research could improve future estimates of overall exposure to NO_2_ and to other pollutants. First, we assessed the contribution of NO_2_ from outdoor sources and from gas and propane stoves. While gas and propane stoves are the overwhelming indoor source of NO_2_, some homes have additional sources of NO_2_ such as ventless space heaters, cigarettes, and gas water heaters and furnaces with improperly functioning flues (53). Second, in modeling range hood use and prevalence we used a nationally representative survey from 1989 (54), California-based surveys from 2012 (55) and 2020 (56), and a Canadian survey from 2023 (57). An updated national survey of range hood prevalence, efficacy, and usage would help improve future exposure estimates. Third, our study focused on residential exposure and we thus did not include potential sources of NO_2_ exposure at work (such as among people working near traffic) or while inside vehicles (58). Fourth, we restricted our analysis to the United States; future work incorporating data on the housing stock, ambient NO_2_, and stove use and ventilation beyond the United States would increase the reach and impact of our model. Fifth, future work could extend our methodology to other pollutants emitted by gas and propane stoves, including benzene (C_6_H_6_), carbon monoxide (CO), formaldehyde (H_2_CO), and ultrafine particles (9, 10, 19, 20, 37–40, 59).

Prior studies quantifying exposure to NO_2_ and its associated disease burden across different geographies typically assume that long-term NO_2_ exposure is equal to the average local outdoor NO_2_ concentrations (2, 3, 13, 50). Our research shows that people with gas and propane stoves receive a substantial portion, in some cases a majority, of their total NO_2_ exposure indoors from fossil fuel combustion by their stoves. With continued improvements in outdoor air quality (36), the relative contribution of indoor NO_2_ to overall NO_2_ exposure is likely to increase even further. Our work can thus inform future estimates of United States and global NO_2_ exposure and disease burden that seek to incorporate both indoor and outdoor sources of NO_2_.

## Materials and methods

### Comparison with previous model of stove-attributable NO_2_ exposure

We previously published an estimate of stove-attributable NO_2_ exposure at the national and state level (12). Like that estimate, this estimate employs CONTAM, a multizone indoor air quality model (see below) (60). Building on the previous estimate, this estimate uses ZIP-code-level input data on dwellings and gas and propane stove prevalence (see below) which result in ZIP-code-level estimates of stove-attributable NO_2_ exposure. This geographic resolution enabled us to incorporate an estimate of exposure due to outdoor sources, thus producing an estimate of total NO_2_ exposure by ZIP code. Since the last work, we have also updated our input assumptions about gas use in stoves and ventilation to better reflect the current state of the literature. These updated assumptions, as well as our use of Census data in this paper as opposed to Residential Energy Consumption Survey data in the last work, resulted in somewhat lower estimates of both stove-attributable and outdoor-attributable NO_2_ exposure. In all cases, the central estimate from each study lies within the other’s 95% CI.

### Sources of outdoor data

We obtained year-averaged outdoor NO_2_ concentrations from 2016 by ZIP code from Columbia University's Socioeconomic Data and Applications Center (45) and hourly outdoor NO_2_ concentrations from the US EPA's network of real-time ground monitors (47). We note that our estimates of outdoor-attributable NO_2_ exposure are limited in remote locations by the prevalence of NO_2_ monitors. We obtained population and population density by ZIP Code Tabulation Area (ZCTA) using data from the 2020 US Census, compiled by Pareto Software, LLC and sold via simplemaps.com (61). We assigned climate designations to ZCTAs based on a given ZCTA's parent county's US Building Climate Zone (62). We assigned ambient temperatures in our model based on average summer and winter averages of a given ZIP code's US Department of Energy Building Climate Zone (62). We assigned weights to different modeled ground windspeeds by drawing from a statistical distribution reported by the National Centers for Environmental Information (63). See Table S2 for more detail.

### Sources of data on dwellings, gas and propane stove prevalence, and occupant behaviors

We used the End-Use Load Profiles (EULP) to derive ZIP-code-level housing stock parameters, including gas and propane stove prevalence. EULP, a comprehensive building energy use database, offers high-granularity and high-quality insights into the US building sector (64). It models ∼550,000 buildings to represent 133.2 million residential dwelling units across the contiguous 48 states of the United States, with detailed housing stock information integrated from multiple government and commercial sources, primarily from around the 2010s (65). Compared with other publicly available regional or state-level data, EULP provides data with substantially higher resolution at the Public Use Microdata Area (PUMA) level. Its accuracy in characterizing US buildings has been validated against actual energy usage data through multidimensional, multivariable comparisons (64).

For each PUMA, we quantified the prevalence (%) of specific home appliances and features, including different types of cooking ranges, central air conditioning and/or heat pump, and categorized floor area (0–1,499, 1,500–2,499, 2,500–3,999, and ≥4,000 ft^2^). We also determined the proportions of different housing types (mobile homes, single family detached homes, single family attached homes, and multifamily homes), and calculated the percentage of single-story and multistory residential units.

We incorporated WorldPop (66), a high-resolution population dataset, to map PUMA-level housing stock parameters to the ZIP-code level. We calculated the average population for each 100 m × 100 m grid cell for the 2010s, which was then used as weights to derive a weighted average of each housing stock parameter for each ZIP-code area.

The source for our distribution of modeled range hood use was Zhao et al. (56) who studied 54 single-family homes and 17 low-income apartments in California in which hood use was monitored using anemometers. Zhao et al. found that hoods are not used 72% of the time in apartments and 64% of the time in houses. We assigned a distribution of different capture efficiencies following a study of 15 commonly installed range hoods representing a spectrum of capture efficiencies (55). Our overall distribution of cooking times was based on Zhao et al. (56) and Klug et al. (67). Our assumptions about the prevalence and efficacy of recirculating hoods in homes with gas stoves come from Sun and Wallace (68; see below for description) and Jacobs and Cornelissen (69), respectively. Jacobs and Cornelissen found that recirculating hoods remove the majority of captured NO_2_ when first installed, but that removal falls off quickly with time and that they remove only 20% of NO_2_ after several weeks. Because NO_2_ removal falls off so quickly with time and because it is uncommon for people to regularly replace filters, we assume that recirculating hoods do not remove NO_2_.

We were unable to find quantitative data on the frequency of burner use at different intensities and therefore relied on other estimates. A 2011 survey of 372 California respondents (67) found that people boiled water (assumed to represent burners on high) in ∼70% of meal preparations, pan- or stir-fried (assumed to represent burners on medium or medium-high) in ∼60% of meals, and simmered (assumed to represent burners on low) in ∼50% of meals. Based on this relatively even spread, we assumed that “on” burners are set to just above medium heat and emit 6.5 megajoules (MJ) of energy per hour. We have previously observed a linear relationship between gas burned and NO_2_ emitted, so we proportionally scaled down the emission rates we measured for burners on high. We thus estimate that on average burners emit 25 mL NO_2_/h (48 mg NO_2_/h), corresponding to half the emission rate of burners on high (Table S2) (10).

As a point of comparison, Chan et al. (70) simulated a relatively intensive meal corresponding to the 75th percentile of gas burned in stoves. This meal produced 100 mg of NO_2_. Meanwhile, our 5th, 50th, and 95th percentile cooking scenarios emit 0.64, 31, and 199 mg of NO_2_ per day on average, respectively.

Our estimates of window opening are based on a survey by Sun and Wallace (68) of 132 homes in Halifax and Edmonton that tracked window opening during and immediately following cooking. To be conservative in our exposure estimate, we assumed that when windows were reported as open, one window was open in the kitchen, one in each bedroom, and one in the living room (3–5 per floorplan, depending on number of bedrooms). In a typical floorplan, assuming instead that an open window corresponds with just the kitchen window being open increases estimated exposure by ∼20% in scenarios where windows are open for a total of 4 h/day (the typical window-open scenario, see Table S2).

We derived our distribution of room occupancy scenarios from the National Human Activity Pattern Survey (43).

The modeled scenarios built from these sources are discussed in more depth in Table S2.

### Overall modeling approach

We used CONTAM, a multizone indoor air model developed by the National Institutes of Standards and Technology, to model indoor NO_2_ concentrations from the stove and percentage infiltration of outdoor NO_2_ (60). CONTAM calculates time-resolved pollutant concentrations separately in each zone (zones correspond with rooms, hallways, stairwells, attic spaces, and other enclosed spaces), then outputs the concentration in each zone in 10-min increments over the course of a modeled 24-h period. Each zone is assumed to be well-mixed, so an output for a given pollutant in a given room at a given time is a single number. Additional assumptions and underlying equations used in CONTAM can be found in chapter 8 of the CONTAM user manual (60).

We previously validated the CONTAM model for estimation of stove-attributable NO_2_ in a set of 18 test houses (12). For this study we modeled exposure in 24 different representative floorplans for each combination of four variables, which we assumed did not change substantively with large-scale geography: range hood use (five values), stove use (three values), room occupancy (five values), and ground windspeed (three values), for a total of 4 × 3 × 5 × 3 = 180 scenarios per floorplan. We assigned normalized weights to the 180 scenarios based on previous research on range hood use, stove use, and occupancy schedules as well as weather data (see Table S2 and the “Sources of data on dwellings, gas and propane stove prevalence, and occupant behaviors” section above). We assumed this distribution remained unchanged between ZIP codes.

Two additional input variables were assumed to be geography dependent: ambient temperature (four values), which varies with climate, and window opening schedules (three values), which we assumed to be a function of ambient temperature and thus indirectly a function of geography (68). We used a prior survey of window opening behavior as a function of ambient temperature (68) to model 2 distributions of exposure for each floorplan corresponding to ambient temperatures of <15 °C, ≥15 °C. Each temperature-dependent exposure distribution thus included 180 × 3 = 540 weighted scenarios.

We applied these NO_2_ exposure distributions to model exposure by ZIP code in the contiguous United States. We used data from the End-Use Load Profiles (64) to produce a dataset reporting various housing stock parameters for each ZIP code. To model exposure in a given ZIP code, we weighted each of our 24 representative floorplans' contribution to the exposure of a given ZIP code based on that ZIP code's Census-derived housing stock parameters. We then assigned each of the 24 floorplans one cold-season and one warm-season exposure distribution (each weighted at 50%) based on the US Department of Energy Building Climate Zone (62) of the ZIP code in question (Table S2). Finally, we adjusted exposure based on cooking times associated with different levels of educational attainment (see below for details).

### Selection and adaptation of the 24 CONTAM floorplans

We selected the 24 floorplans used in this study from a pool of 209 dwellings constructed in CONTAM by Persily et al. (71) to represent the US housing stock. The 24 floor plans represent diverse yet common types of homes that encompass the characteristics of the US housing stock (see Table S1 for a summary of the 24 floorplans). These 24 floorplans are the same 24 we used in a previous study (12). We assigned floorplan weights in each ZIP code according to the relative prevalence of different home types, ranges of floor area (i.e. square footage), number of stories, presence or absence of a forced-air system, and ranges of home age as described previously (12).

We left each model floorplan taken from Persily et al.'s (71) collection unchanged except for the modifications we described previously (12). Briefly, we modified Persily et al.'s floorplans by adding one National Fenestration Research Council standard window (1.2 m by 1.5 m or 4 ft. by 5 ft.) (56) to an exterior wall in every bedroom, living room, and kitchen and replacing interior doors with fixed bi-directional airflow elements. We left central forced-air systems unchanged and assigned their schedule based on modeled ambient temperature (Table S2).

We also made changes to better model range hoods. Because CONTAM models each zone as well-mixed, CONTAM interprets modeled exhaust fans as removing a given flowrate of well-mixed air. Because the air removed by range hoods is not yet well-mixed, exhaust fans are poor approximations of range hoods. To better model the effect of a range hood, we removed the modeled kitchen exhaust fan and instead multiplied the stove's emission rate by a capture efficiency coefficient (for instance, we modeled a 50% capture efficiency hood by multiplying the stove's emission rate by 0.5).

We note that in principle removing the kitchen exhaust fan may alter the model because in addition to removing pollution from the stove, the exhaust fan also removes well-mixed kitchen air. However, this impact on long-term and acute exposure is low. We modeled kitchen NO_2_ concentrations in MH-1, a small floorplan, under the high stove use scenario with and without the kitchen exhaust fan. We found average and max hour-averaged kitchen concentrations to be 2.7 and 4.2% higher, respectively, without the exhaust fan than with the exhaust fan. This floorplan and scenario combination would be most sensitive to the hood removal. The actual impact on long-term and acute exposure will be far lower than this since most floorplans are larger, most scenarios involve less stove use, hood use is infrequent, and people spend most of their time in other rooms where the impact of the additional removal will be smaller.

### Identification of input variables

We captured a range of behaviors and environments by modeling stove-attributable and outdoor-attributable NO_2_ exposure in each of our 24 selected floorplans for three to five values for each of six parameters: range hood use, stove use, room occupancy, ground windspeed, window opening schedule, and ambient temperature. We modeled the same values of these parameters as in our previous work (12), except for room occupancy. Here, we included two additional occupancy schedules representing the 5th and 95th percentiles of time spent outdoors to capture a wider range of time spent outdoors than in our previous study.

Table S2 defines our choice of values for each variable as well as their assigned normalized weights. For each floorplan we modeled 4 × 3 × 3 × 5 × 3 × 4 = 2,160 scenarios for a total of 51,840 scenarios.

### Calculating stove- and outdoor-attributable NO_2_

We assumed that indoors, NO_2_ decays at the previously reported rate of −2.4 × 10^−4^ s^−1^ (-0.86 h^−1^) (72). We included literature-reported decay rates ranging from −4.7 × 10^−5^ s^−1^ (−0.17 h^−1^) to −5.7 × 10^−4^ s^−1^ (−2.1 h^−1^) in our uncertainty estimate (see below). We estimated stove-attributable NO_2_ exposure directly by modeling NO_2_ emissions from gas and propane stoves indoors. We modeled outdoor-attributable NO_2_ exposure using a dummy pollutant, which we called CONTA, with identical physical properties to NO_2_. We set the outdoor concentration of CONTA to a constant value of 100 ppbv so that it is converted into a percentage. The concentration of CONTA in a given room in ppbv can thus be interpreted as a percent infiltration. We thus calculated outdoor-attributable NO_2_ exposure as


$$OA_{NO_{2}}=OA_{CONTA}\times \frac{\left[NO_{2}\right]}{\left[CONTA\right]}$$

where $OA_{NO_{2}}$ is the outdoor-attributable NO_2_ exposure, $OA_{CONTA}$ is the modeled exposure to the dummy pollutant CONTA in ppbv, $\left[NO_{2}\right]$ is the outdoor concentration of NO_2_ in ppbv, and [CONTA] is the outdoor concentration of CONTA, set to 100 ppbv. Using the dummy pollutant CONTA allowed us to cut down on computation by modeling the percentage infiltration of NO_2_ for each floorplan and combination of input variables and then to apply that infiltration percentage to different local outdoor NO_2_ concentrations, rather than re-running the CONTAM model for every outdoor concentration of NO_2_ in every location.

### Converting 24-h concentration time courses into reported long- and short-term exposures

We converted a given 24-h exposure scenario (see Supplementary Definitions) into its corresponding long- and short-term exposure values as follows:


(1) $$\mathrm{Ex}p_{\mathrm{chro}n_{\mathrm{day}}}=\frac{\Sigma_{\left\{i=1\right\}}^{144}\;\;C_{i}}{144}$$

(2) $$\mathrm{Ex}p_{\mathrm{hour}}(i)=\frac{C_{i+5}-C_{i}}{6}$$

(3) $$\mathrm{Ex}p_{\mathrm{acut}e_{\mathrm{day}}}=\underset{i\in \;Z\;\;:\;i\in [1,\;144]}{\max}\;(\mathrm{Ex}p_{\mathrm{hour}}(i))$$

where $\mathrm{Ex}p_{\mathrm{chro}n_{\mathrm{day}}}$ is the modeled mean long-term exposure to a pollutant assuming the modeled 24-h period is repeated indefinitely, $C_{i}$ is the 10-min-averaged concentration at interval *i* (there are 144 such 10-min intervals in a 24 h day), and $\mathrm{Ex}p_{\mathrm{acut}e_{\mathrm{day}}}$ is the maximum hour-averaged exposure during the modeled day. The pollutant may be NO_2_ or CONTA, the dummy pollutant used to estimate outdoor-attributable NO_2_ exposure. We then weight $\mathrm{Ex}p_{\mathrm{chro}n_{\mathrm{day}}}$ and $\mathrm{Ex}p_{\mathrm{acut}e_{\mathrm{day}}}$ based on their weekly prevalence (Table S2). For instance, the three different values of $\mathrm{Ex}p_{\mathrm{chro}n_{\mathrm{day}}}$ corresponding to the three different day scenarios associated with median stove use would be weighted as 2/7, 4/7, and 1/7, respectively, to calculate a scenario-specific exposure value $\mathrm{Ex}p_{\mathrm{chron}}$. We performed an analogous calculation for $\mathrm{Ex}p_{\mathrm{acute}}$.

Because weighting the 95th percentile for short-term exposure exceedances at 10% may bias our estimate high, we also calculated the number of exceedances if we adjusted our weighting to 5%. We report the resulting range of days with an exceedance in the results.

### Calculating ZIP code-, county-, and national NO_2_ exposures

To calculate exposure in a given ZIP code, we assigned normalized weights to each of our 24 model floorplans based on that ZIP code's housing stock parameters (see Fig. S1 for a decision flow chart and more detailed description of this assignment). We selected one of the nine temperature-specific exposure distributions based on that ZIP code's Building America Climate Zone (62) and corresponding window opening behavior (see Table S2 under “Window Use” and “Ambient Temperature”). Each floorplan's exposure distribution thus contained 180 normalized weighted exposure scenarios (see “Overall modeling approach” section).


(4) $$\begin{array}{r} \mathrm{Ex}p_{\mathrm{chro}n_{\mathrm{ZIP}}}=\frac{\Sigma_{\left\{i=1\right\}}^{24}\Sigma_{\left\{\;j=1\right\}}^{180}\mathrm{Ex}p_{\mathrm{chro}n_{\left\{i,j\right\}}}\times \mathrm{WScenari}o_{j}\times \mathrm{WFloorpla}n_{\left\{i,\mathrm{ZIP}\right\}}}{180\times 24} \end{array}$$

(5) $$\begin{array}{r} \mathrm{Ex}p_{\mathrm{acut}e_{\mathrm{ZIP}}}=\frac{\Sigma_{\left\{i=1\right\}}^{24}\Sigma_{\left\{\;j=1\right\}}^{180}\mathrm{Ex}p_{\mathrm{acut}e_{\left\{i,j\right\}}}\times \mathrm{WScenari}o_{j}\times \mathrm{WFloorpla}n_{\left\{i,\mathrm{ZIP}\right\}}}{180\times 24} \end{array}$$

(6) $$f(\mathrm{conc},\;\mathrm{threshold})=\;\{\begin{array}{c} 0\;\;\;\;\;\mathrm{conc}<\mathrm{threshold}\\ 1\;\;\;\;\;\mathrm{conc}\geq \mathrm{threshold} \end{array}$$

(7) $$\mathrm{Excee}d_{\mathrm{chron}\mathrm{ZIP}}=\;\frac{\Sigma_{\left\{i=1\right\}}^{24}\Sigma_{\left\{\;j=1\right\}}^{180}f(\mathrm{Ex}p_{\mathrm{chron}},\;\mathrm{threshold})\times \mathrm{WScenari}o_{j}\times \mathrm{WFloorpla}n_{\left\{i,\mathrm{ZIP}\right\}}}{180\times 24}\;$$

(8) $$\mathrm{Excee}d_{\mathrm{acute}\mathrm{ZIP}}=\;\frac{\Sigma_{\left\{i=1\right\}}^{24}\Sigma_{\left\{\;j=1\right\}}^{180}f(\mathrm{Ex}p_{\mathrm{acute}},\;\;\mathrm{threshold})\times \mathrm{WScenari}o_{j}\times \mathrm{WFloorpla}n_{\left\{i,\mathrm{ZIP}\right\}}}{180\times 24}\;$$

where $\mathrm{Ex}p_{\mathrm{chro}n_{\mathrm{ZIP}}}$ is the mean long-term pollutant exposure in a given ZIP code, $\mathrm{Ex}p_{\mathrm{acut}e_{\left\{i,j\right\}}}$ is the mean exposure for the *i*th floorplan in the *j*th exposure scenario, $\mathrm{Ex}p_{\mathrm{acut}e_{\mathrm{ZIP}}}$ is the mean maximum daily hour-averaged exposure in a given ZIP code, $\mathrm{Ex}p_{\mathrm{acut}e_{i,j}}$ is the maximum hour-averaged exposure for the *i*th floorplan in the *j*th exposure scenario, all for people with gas or propane stoves, and $\mathrm{WScenari}o_{j}$ is the weight of the *j*th exposure scenario, $\mathrm{WFloorpla}n_{\left\{i,\mathrm{ZIP}\right\}}$ is the weight of the *i*th floorplan for a given ZIP code, and $\mathrm{Excee}d_{\mathrm{chro}n_{\mathrm{ZIP}}}$ and $\mathrm{Excee}d_{\mathrm{acut}e_{\mathrm{ZIP}}}$ are the fractions of long-term and short-term exposures, respectively, in a given ZIP code that exceed a given threshold.

To calculate the equivalent exposure parameter across the entire population of ZIP code, including people with electric coil and induction stoves, we divided the exposure parameter for people with gas and propane stoves by the fraction of the total population with gas or propane stoves.

We calculated population-weighted long- and short-term exposures for larger geographies such as counties and the contiguous United States as:


(9) $$\mathrm{Exposure}=\frac{\Sigma_{\mathrm{allZIPs}}\mathrm{Exposur}e_{i}\times \mathrm{Populatio}n_{i}}{\Sigma_{\mathrm{allZIPs}}\mathrm{Populatio}n_{i}\;}\;$$

where Exposure is the desired population-averaged exposure value (long-term, short-term, or short-term threshold exceedances) in a given geography, allZIPs is the set of all ZIP codes within the given geography, $\mathrm{Exposur}e_{i}$ is the desired exposure value in the *i*th ZIP code, and $\mathrm{Populatio}n_{i}$ is the population of the *i*th ZIP code.

#### Air exchanges

We calculated modeled air exchanges using a dummy pollutant, CONTB. We calculated the air exchange constant associated with a given exposure scenario as k=1/τ, where *τ* is the time constant representing the time required for the interior concentration of CONTB to equal 1−(1/e) (∼63.2%) of the outdoor concentration. We calculated a mean air exchange constant across the country of 0.74 h^−1^. Values for individual scenarios ranged from 0 h^−1^ (windows closed, equal indoor and outdoor temperatures, no wind) to 6.0 h^−1^ (windows open, outdoor temperature = −5 °C, 10 m/s wind). As a point of comparison, these values generally agree with those obtained by Murray and Burmaster, who surveyed 2,844 residences throughout the United States and calculated a mean air exchange of 0.76 h^−1^ with individual values ranging from 0.01 to 11.77 h^−1^ (73).

### Uncertainty

To calculate uncertainty in exposure in each ZIP code, we computed a Monte Carlo distribution resulting from the combination of five input distributions representing five key elements of the model: (i) measured NO_2_ emission factors, (ii) the NO_2_ decay rate, (iii) estimated burner intensities used, (iv) the distribution of the ratios of NO_2_ concentrations modeled by CONTAM with those directly measured in 18 test houses we previously analyzed (12), and (5) the distribution of cooking times measured by Zhao et al. (56), using Eqs. 10a and 10b. We then calculated the 2.5th, 50th, and 97.5th percentiles of each distribution and divided these by the distribution's median such that the scaled median equaled one. We then multiplied our estimated day-averaged and maximum hour-averaged exposure for each modeled scenario by these values to obtain the 95% CI for hour-averaged and day-averaged exposure associated with each of our modeled scenarios. This method assumes that the relationship between the input parameters and exposure does not deviate extremely from linearity. Future work could further constrain our estimates by running full Monte Carlo simulations through CONTAM or by developing and testing nonlinear analytical relationships between these input parameters and exposure.


(10a ) $$MC_{\mathrm{stove}}=\mathrm{mean}(\mathrm{EF}\times \mathrm{DR}\times \mathrm{BI}\times (1-\mathrm{CE})\times U)$$

(10b ) $$MC_{\mathrm{outdoor}}=\mathrm{mean}(\mathrm{DR}\times (1-\mathrm{CE}))$$

where *EF*, *DR, BI*, *CE,* and *U* are randomly sampled (with replacement) values from the distributions of emission factors, NO_2_ decay rates, burner intensities, ratios of modeled to measured exposures in test houses, and minutes of cooktop use measured. We modeled the distribution of decay rates as a skewed normal distribution centered on −2.4 × 10^−4^ s^−1^ (−0.86 h^−1^) where 95% of values fall between −4.7 × 10^−5^ s^−1^ (−0.17 h^−1^) and −5.7 × 10^−4^ s^−1^ (−2.07 h^−1^). The central estimate corresponds with the mean value reported in a partially carpeted house while the lower and upper bounds correspond with the lower and upper average values reported in the literature (72).

Because we have relatively sparse data on the frequency with which people cook with burners on low, medium, and high intensities (56), we assumed a normal distribution for burner intensity expressed as a fraction of joules burned per unit time on high (mean = 0.5; standard deviation = 0.1). We calculated MC_stove_ and MC_outdoor_ over 10,000 iterations to form a Monte Carlo distribution and calculated 95% CIs for exposure based on the Monte Carlo distributions.

We calculated central estimate, lower bound, and upper bound long-term exposure for each floorplan as the average of the central estimate, lower bound, and upper bound of modeled day exposures, respectively. We calculated short-term exposure exceedances as the fraction of modeled days with a maximum 1-hour average exposure exceeding 100 ppbv and converted the fraction to days per year by multiplying by 365. We used the central estimate, lower bound, and upper bound modeled day exposures to calculate our CI in an analogous fashion to long-term exposures.

We calculated lower bounds, central estimates, and upper bounds of summary exposure statistics (which include multiple ZIP codes) as follows:


(11a ) $$\begin{array}{r} f_{\mathrm{Lower}}(\mathrm{ZI}P_{1},\;\mathrm{ZI}P_{2},\ldots ,\;\mathrm{ZI}P_{n})=f(\mathrm{ZI}P_{1\mathrm{Lower}},\;\mathrm{ZI}P_{2\mathrm{Lower}},\ldots ,\mathrm{ZI}P_{n_{Lower}}) \end{array}$$

(11b ) $$\begin{array}{r} f_{\mathrm{Central}}(\mathrm{ZI}P_{1},\;\mathrm{ZI}P_{2},\ldots ,\mathrm{ZI}P_{n})=f(\mathrm{ZI}P_{1\mathrm{Central}},\;\mathrm{ZI}P_{2\mathrm{Central}},\ldots ,\mathrm{ZI}P_{n_{\mathrm{Central}}}) \end{array}$$

(11c ) $$\begin{array}{r} f_{\mathrm{Upper}}(\mathrm{ZI}P_{1},\;\mathrm{ZI}P_{2},\ldots ZIP{,}_{n})=f(\mathrm{ZI}P_{1\mathrm{Upper}},\;\mathrm{ZI}P_{2\mathrm{Upper}},\ldots ,\mathrm{ZI}P_{n_{Upper}}) \end{array}$$

where $f_{\mathrm{Lower}}$, $f_{\mathrm{Central}}$, and $f_{\mathrm{Upper}}\;$ return the lower bound, central estimate, and upper bound, respectively, of the result of function *f* applied *n* ZIP codes and where $\mathrm{ZI}P_{i\mathrm{Lower}}$, $\;\mathrm{ZI}P_{i\mathrm{Central}}$, and $\mathrm{ZI}P_{i_{\mathrm{Upper}}}$ are the lower bound, central estimate, and upper bound of the estimated NO_2_ exposure in ZIP code *i*, where *i* runs through all ZIP codes included in the summary statistic in question. For example, we would report the lower bound of the average exposure of the three ZIP codes Z1, Z2, and Z3 as the average of the lower bounds Z1, Z2, and Z3.

## Supplementary Material

pgaf341_Supplementary_Data

## Data Availability

All data used in this analysis beyond those presented in the main text and Supplementary Material is available on the Zenodo repository associated with this manuscript (10.5281/zenodo.11204957). All codes used in this analysis are available on the Zenodo repository associated with this manuscript (10.5281/zenodo.11204957). Re-running the analysis requires downloading the software packages CONTAMW and CONTAMX (available for download at https://www.nist.gov/el/energy-and-environment-division-73200/nist-multizone-modeling/software/contam/download), installing JupyterLab (available for installation at https://jupyter.org/), and installing Python 3 (version 3.12.2 used in this analysis; available for installation at https://www.python.org/downloads/).
