# Supplementary material for: Integrating indoor and outdoor nitrogen dioxide exposures in US homes nationally by ZIP code
Source: PNAS Nexus. 2025 Dec 2;4(12):pgaf341. doi: 10.1093/pnasnexus/pgaf341 (PMC12670381; doi:10.1093/pnasnexus/pgaf341)
Supplement: pgaf341_Supplementary_Data [file pgaf341_supplementary_data.zip › PNASNEXUS-PNASNEXUS-2025-00244-TRR-s01.pdf]

PNAS nexus

Supplementary Information for

Integrating Indoor and Outdoor Nitrogen Dioxide Exposures in U.S. Homes Nationally by ZIP Code

Authors: Yannai Kashtan[1,2], Chenghao Wang[3,4], Kari C. Nadeau[5], Robert B. Jackson[1,6]*

Corresponding author: Rob.Jackson@stanford.edu

This Documents file includes:

Supplementary text
Definitions
Figures S1 to S19
Tables S1 to S3
Legends for Datasets S1 and S2
SI References

Other supplementary materials for this manuscript include the following:

Datasets S1 and S2

Supplementary Information Text

Definitions

We define a "cooktop" as a flat surface with 2-6 individual cooking elements and "burners" as cooking elements using a gas or propane flame. We define gas, propane, and electric ovens as enclosed spaces heated by gas, propane, or electricity, respectively. We define a "stove" (also called a "range") as a freestanding unit that contains both a cooktop and an oven. We define an "outside-venting range hood" as a mechanical ventilation unit that draws indoor air outside located directly above a stove, cooktop, or oven used to remove pollutants produced while cooking. We define a "recirculating range hood" as a range hood that returns exhaust air to the kitchen rather than venting it outdoors.

Throughout the paper we use the term "concentration" for its accessibility in place of the more strictly correct term "molar mixing ratios." We assume a temperature of 25 ºC and a pressure of 1 atm when converting between true concentrations and molar mixing ratios, which yields the conversion 1 ppbv NO_2 = 1.89 μg m^{-3} NO_2.

We define "stove-attributable" NO_2 exposure as NO_2 exposure due to NO_2 emitted from a stove. We define "outdoor-attributable" NO_2 exposure as NO_2 exposure attributable to NO_2 from all outdoor sources, whether breathed outdoors or breathed indoors when outdoor pollution infiltrates the home.

We define "infiltration" as the process of outdoor pollutants entering homes, typically via incomplete weatherization, through HVAC systems, or through open doors or windows. "Infiltration fraction" refers to the ratio of the concentration of a pollutant measured indoors, in the absence of indoor sources of the pollutant, to the concentration of the pollutant outdoors. "Infiltration percentage" refers to the same quantity expressed as a percentage.

Concentration vs. exposure: We define a "modeled concentration" as the modeled NO_2 concentration in a given room of a dwelling or in the ambient air at a given time, irrespective of the modeled occupancy schedule. In contrast, we define a "modeled exposure" as the NO_2 concentration a modeled occupant is exposed to averaged over a given time interval, given a set occupancy schedule. Both modeled concentrations and modeled exposures are reported in ppbv.

We define an "exposure scenario" as a single, 24-hour-long modeled exposure timecourse in a given dwelling, for a given level of range hood use and stove use, a given occupancy schedule, a given ground windspeed outdoors, and a given ambient temperature. Realtime exposure is modeled in 5 second increments and reported in 10-minute increments.

Throughout the manuscript, we use the term "ZIP codes" for its accessibility in place of the more strictly correct "ZIP Code Tabulation Areas" (ZCTAs). ZCTAs are areal representations of point-based postal ZIP codes developed by the U.S. Census Bureau for geographic analysis (*1*). Because our analysis includes Census data available only for ZCTAs, we restricted our analysis to the ZCTAs reported in the 2020 Census.

We defined “urban ZIP codes” as ZCTAs in the top 90th percentile of population density and “rural ZIP codes” as ZCTAs in the bottom 10th percentile of population density.

We define “gas stove use intensity” as the amount of gas burned in the stove. The 95th percentile of gas stove use corresponds to having two burners on medium for 41 minutes in the morning, four burners on medium for 41 minutes in the evening, and the oven set to 350ºF for 2 hours and 14 minutes in the evening (approximately 64 megajoules of gas burned per day) (*2*). The 50th percentile of gas stove use corresponds to having one burner on medium for 30 minutes in the morning, two burners on medium for 30 minutes in the evening, and no oven (approximately 10 megajoules of gas burned per day) (*2*). The 5th percentile of gas stove use corresponds to having one burner on medium for 5 minutes in the morning, two burners on medium for 5 minutes in the evening, and no oven (*2*).

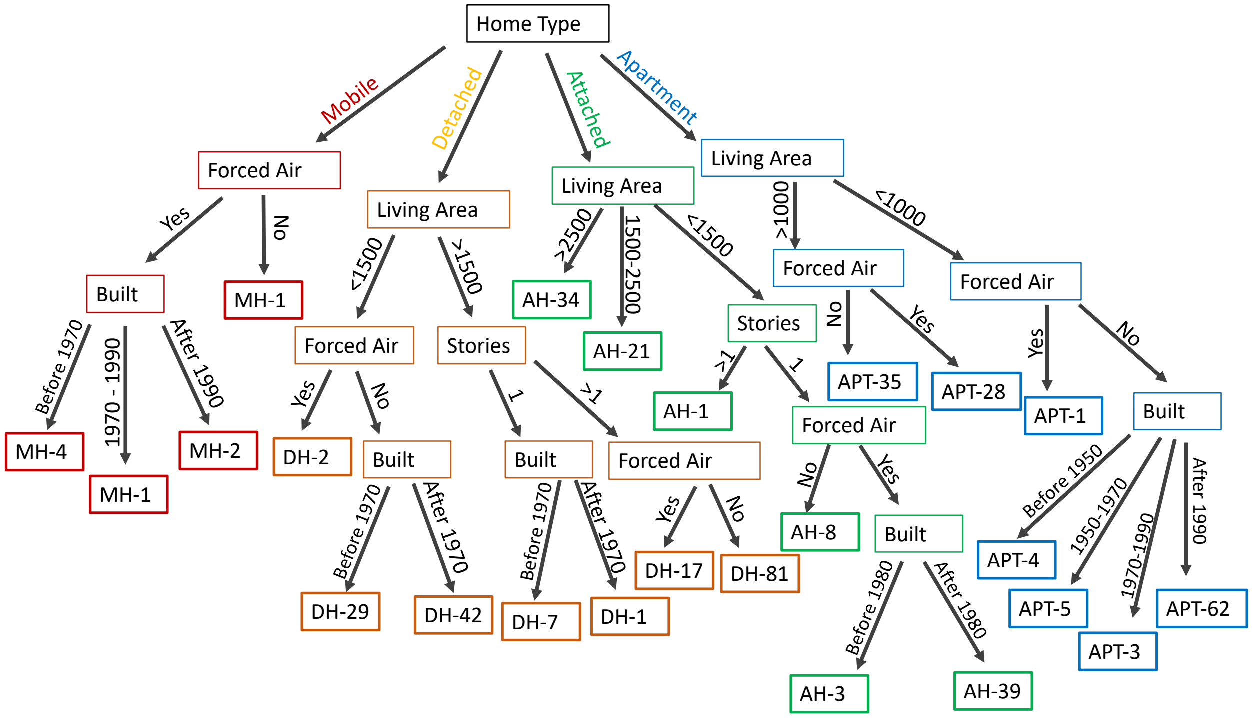

Fig. S1

Flowchart used in assigning CONTAM floorplans produced by Persily et al. *(3)*. "Forced air" refers to the presence or absence of a central forced-air system in the house. Living area is reported in square ft. Listed floorplan codes are as used by Persily et al. These floorplans were previously used to estimate NO_2 exposure attributable to gas and propane stoves *(4)*.

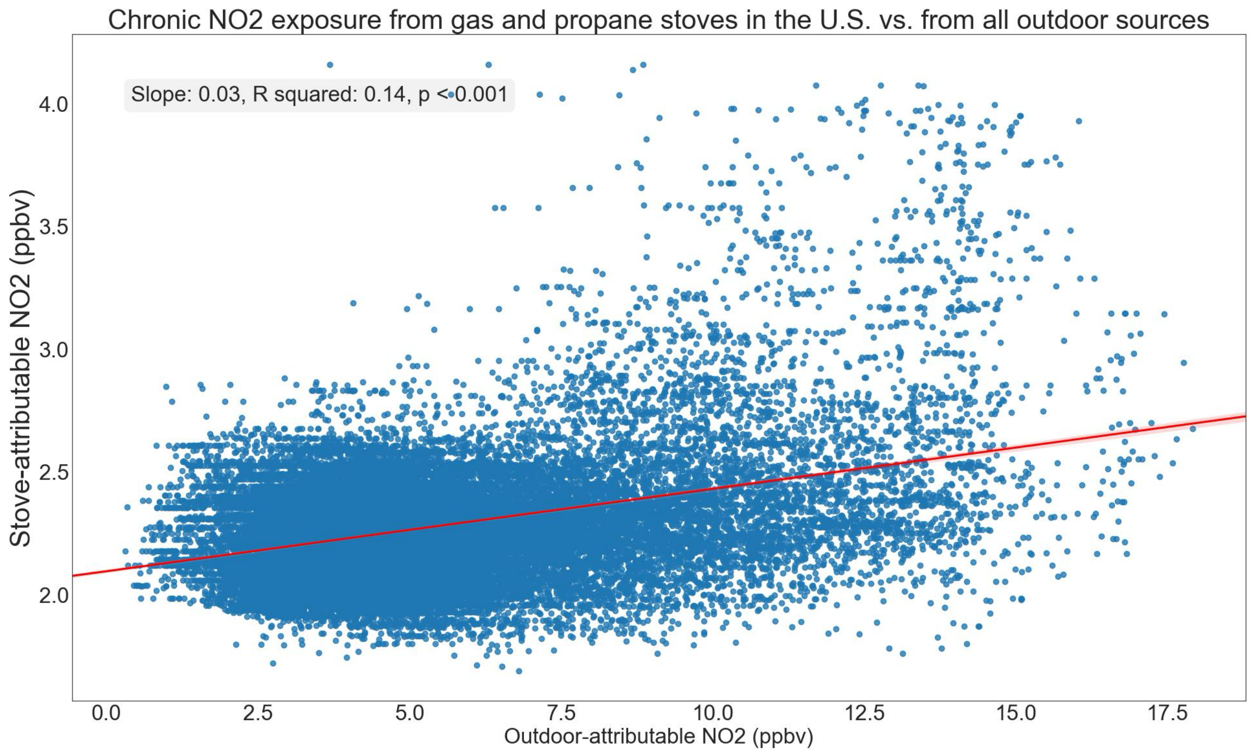

Fig. S2. Ordinary linear regression of stove-attributable vs. outdoor-attributable chronic NO_2 exposure by ZIP code across the contiguous U.S. (Slope = 0.03, $r^2 = 0.14$, $p < 0.001$).

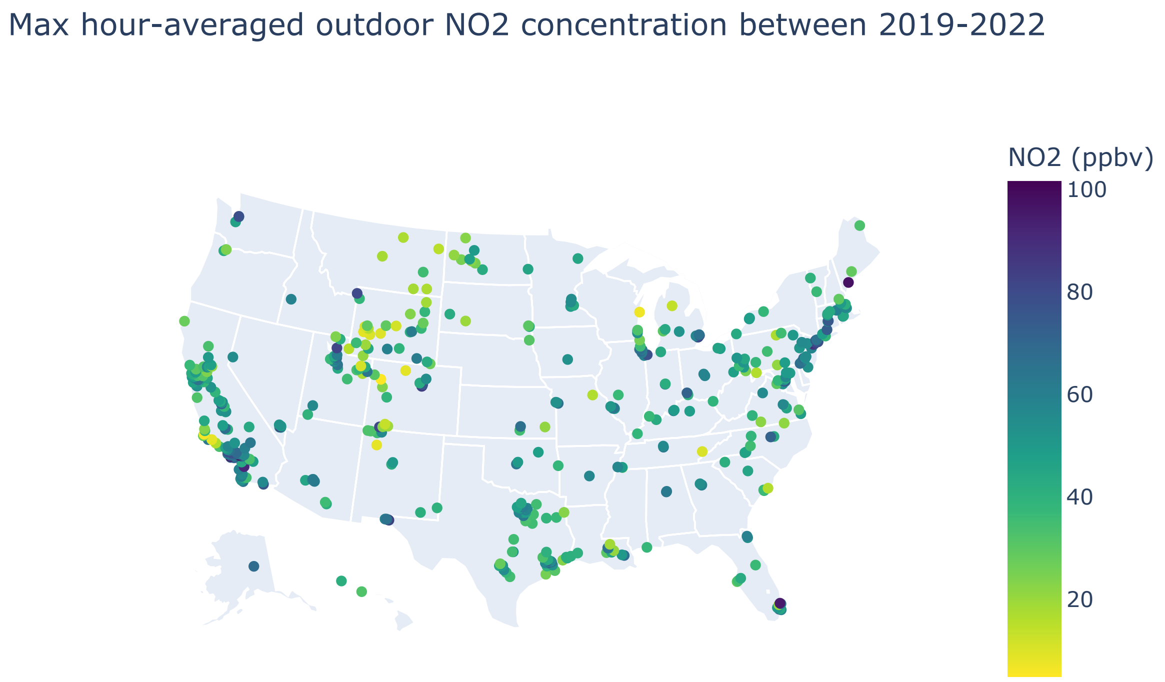

Fig. S3. Maximum hour-averaged outdoor NO_2 concentrations recorded by US EPA ground-level sensors between 2019-2022, inclusive (*5*).

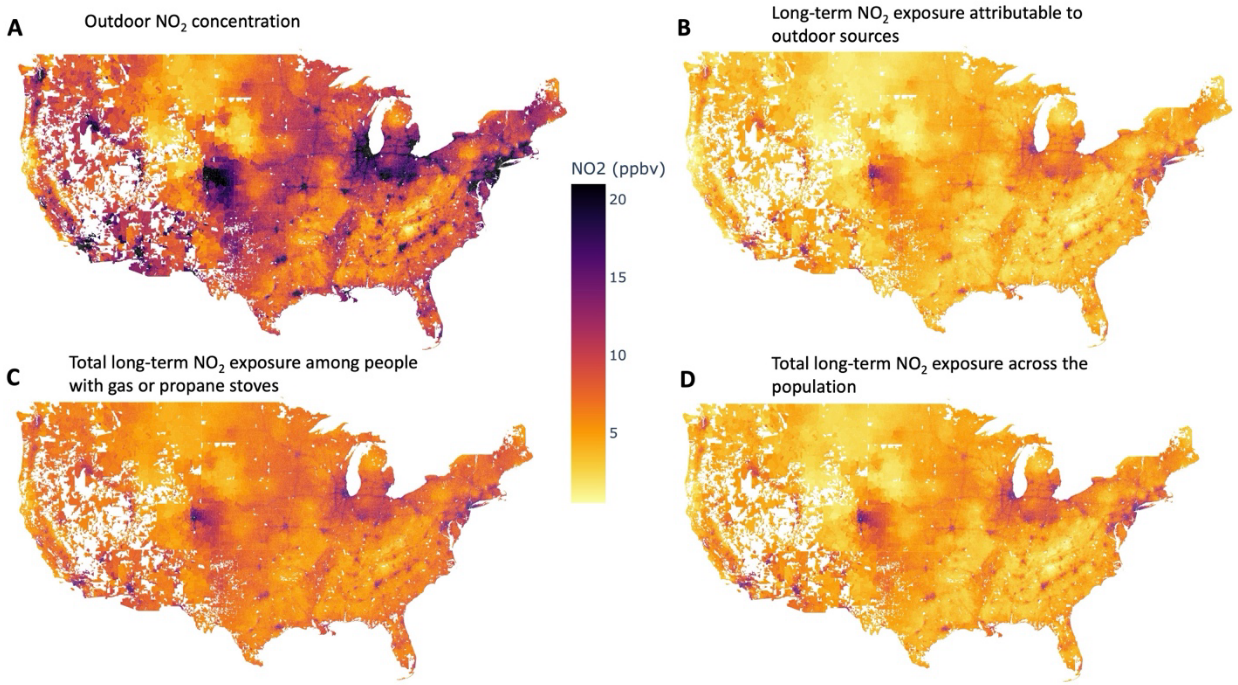

Fig. S4 (A) Outdoor NO_2 concentrations (a reproduction of Columbia's SEDAC model) (*6*) and our estimates of long-term NO_2 exposure attributable **(B)** to outdoor sources, **(C)** to outdoor sources plus gas and propane stoves among households who have them, and **(D)** to outdoor sources plus gas and propane stoves among the entire population including households with electric cooking. "Total" exposure refers to total exposure within the constructs of our model and does not include occupational NO_2 exposures. Gaps represent locations lacking ZIP codes. Note that subplot B is identical to Fig. 2A in the main manuscript. It is reproduced here for ease of comparison.

Figures S5 – S18 and Table S3 report modeled NO_2 exposure values for New York City, San Francisco, CA, Los Angeles, CA, Washington, D.C., Denver, CO, Houston, TX, and Bakersfield, CA. We chose these cities because they represent a diversity of climate and urban form and because these are the cities in which we previously measured NO_2 emission rates from gas and propane stoves .

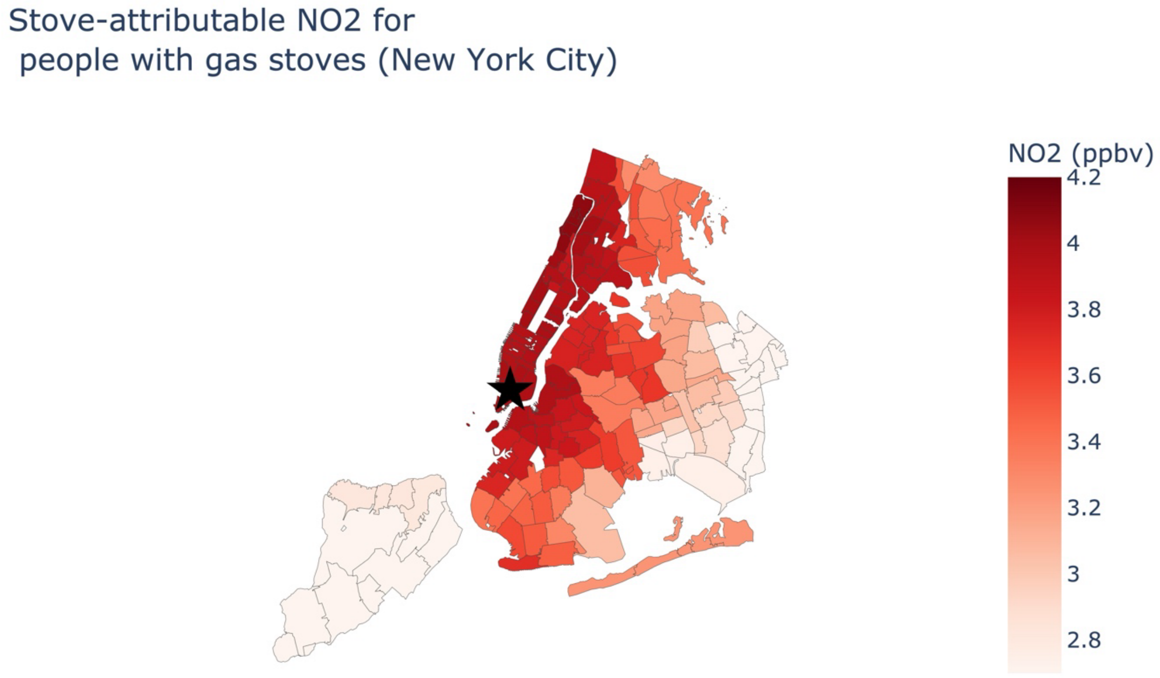

Fig. S5. Modeled stove-attributable long-term NO_2 exposure for people with gas or propane stoves in New York, NY. Black dots indicate the central business district or downtown of each metro area.

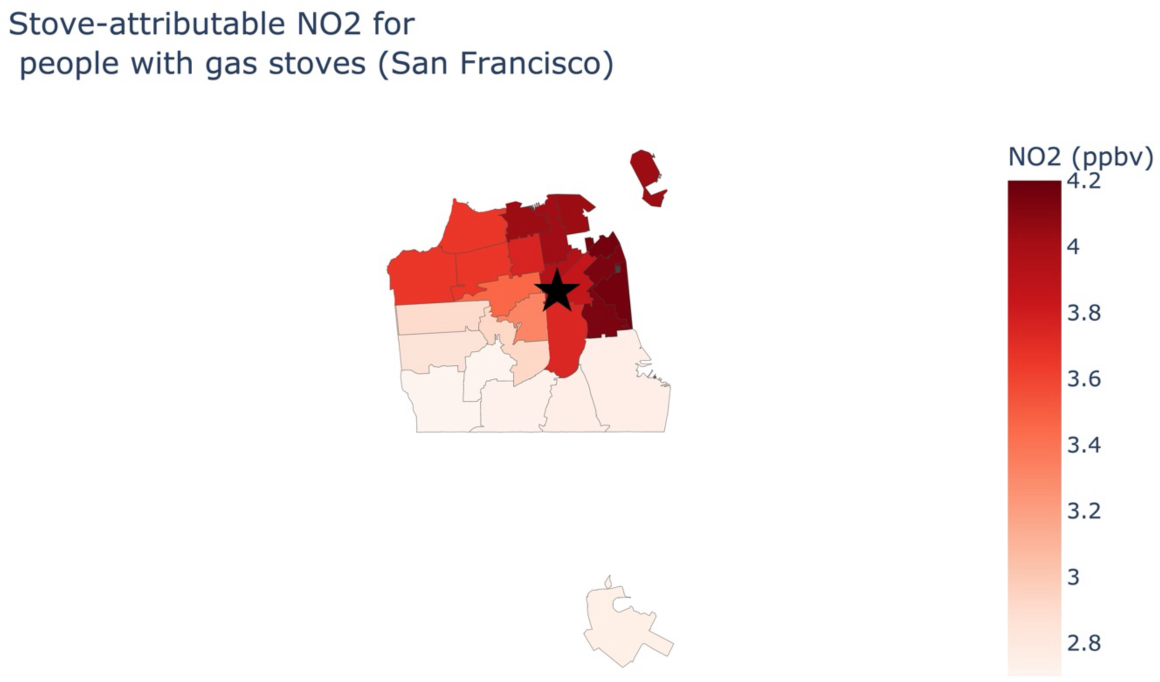

Fig. S6. Modeled stove-attributable long-term NO_2 exposure for people with gas or propane stoves in San Francisco, CA. The black star indicates downtown San Francisco.

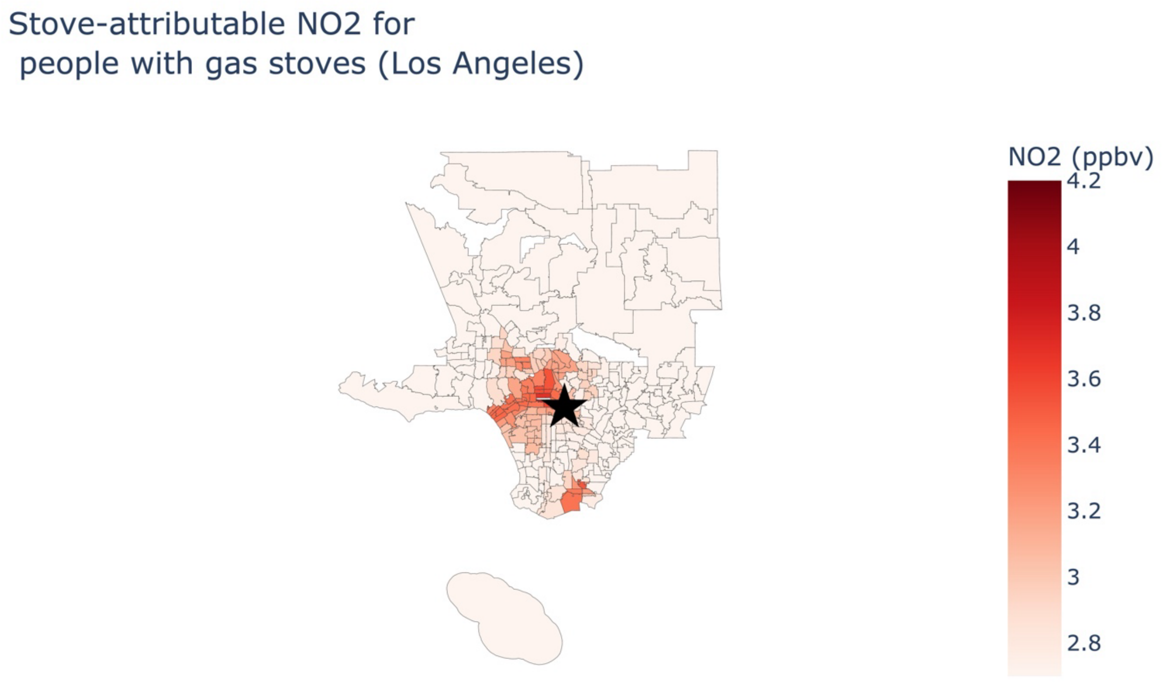

Fig. S7. Modeled stove-attributable long-term NO_2 exposure for people with gas or propane stoves in Los Angeles county, CA. The black star indicates downtown Los Angeles.

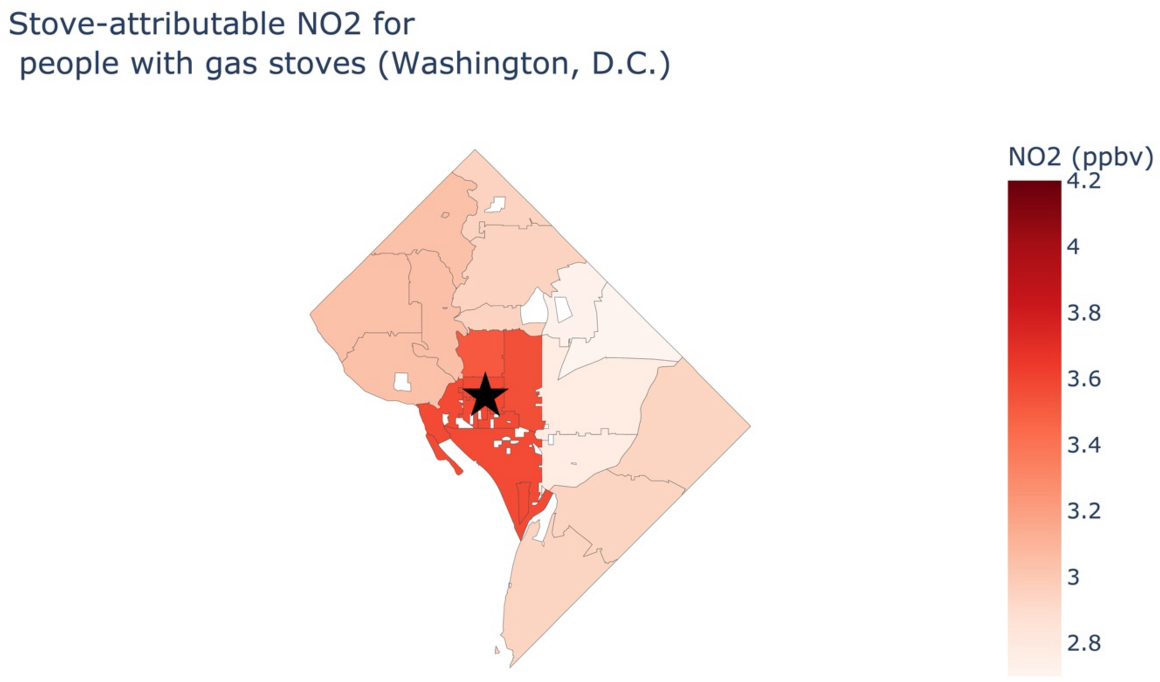

Fig. S8. Modeled stove-attributable long-term NO_2 exposure for people with gas or propane stoves in Washington, D.C. The black star indicates downtown.

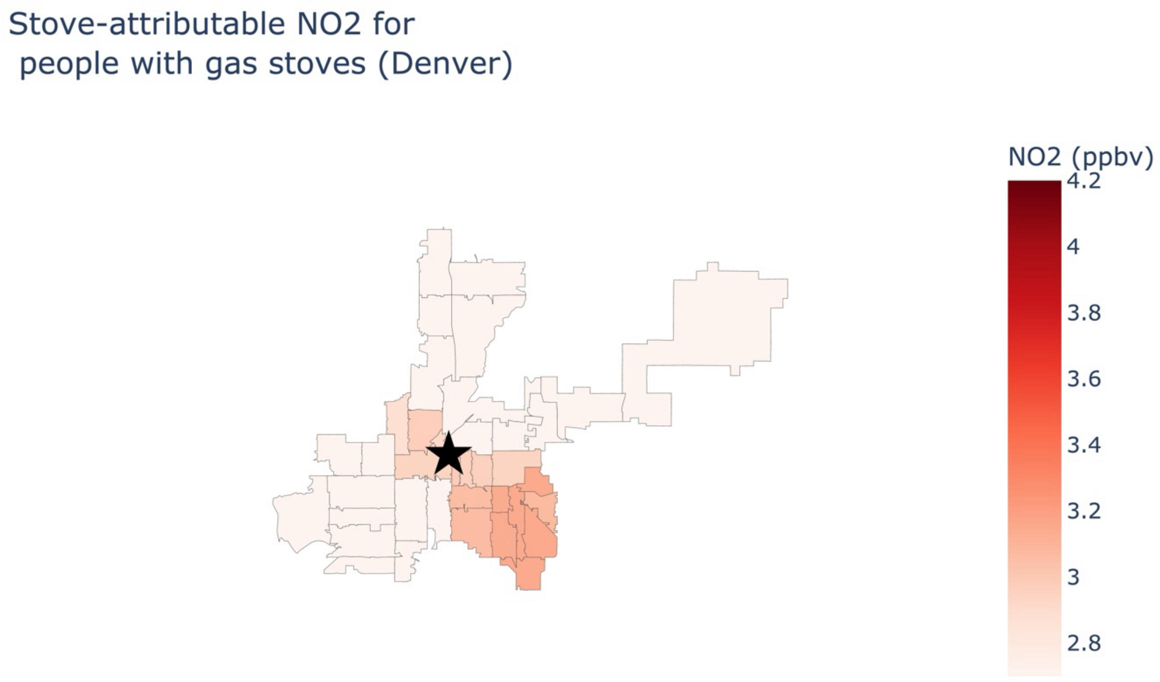

Fig. S9. Modeled stove-attributable long-term NO_2 exposure for people with gas or propane stoves in Denver, CO. The black star indicates downtown.

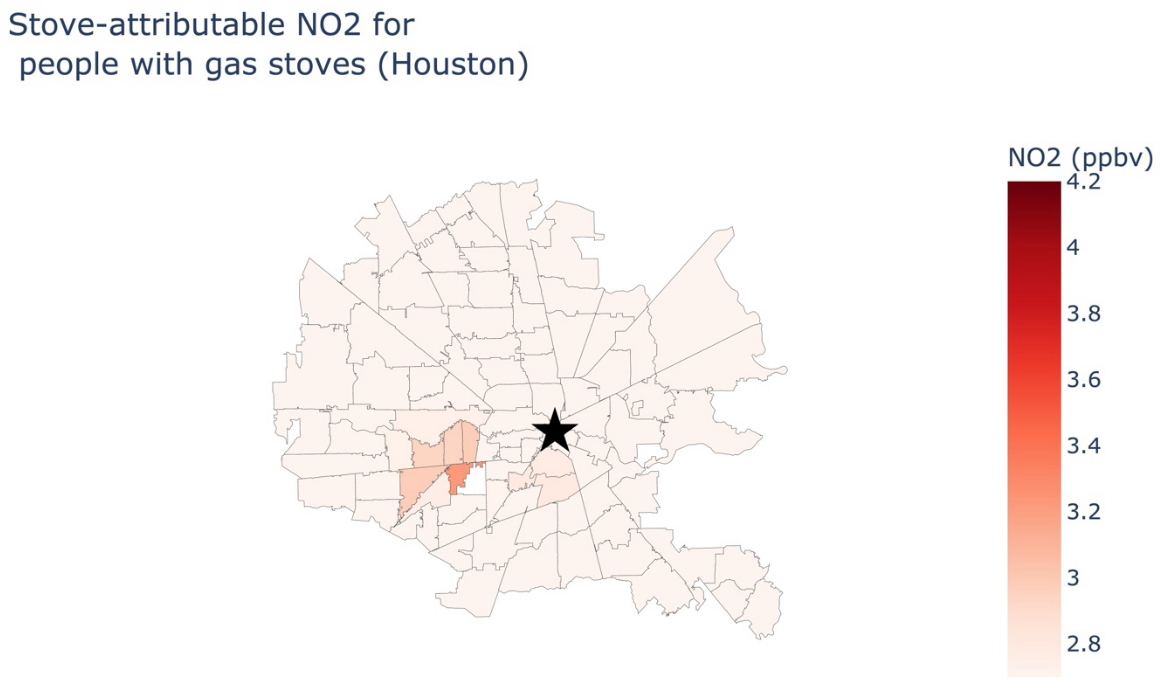

Fig. S10. Modeled stove-attributable long-term NO_2 exposure for people with gas or propane stoves in Houston, TX. The black star indicates downtown.

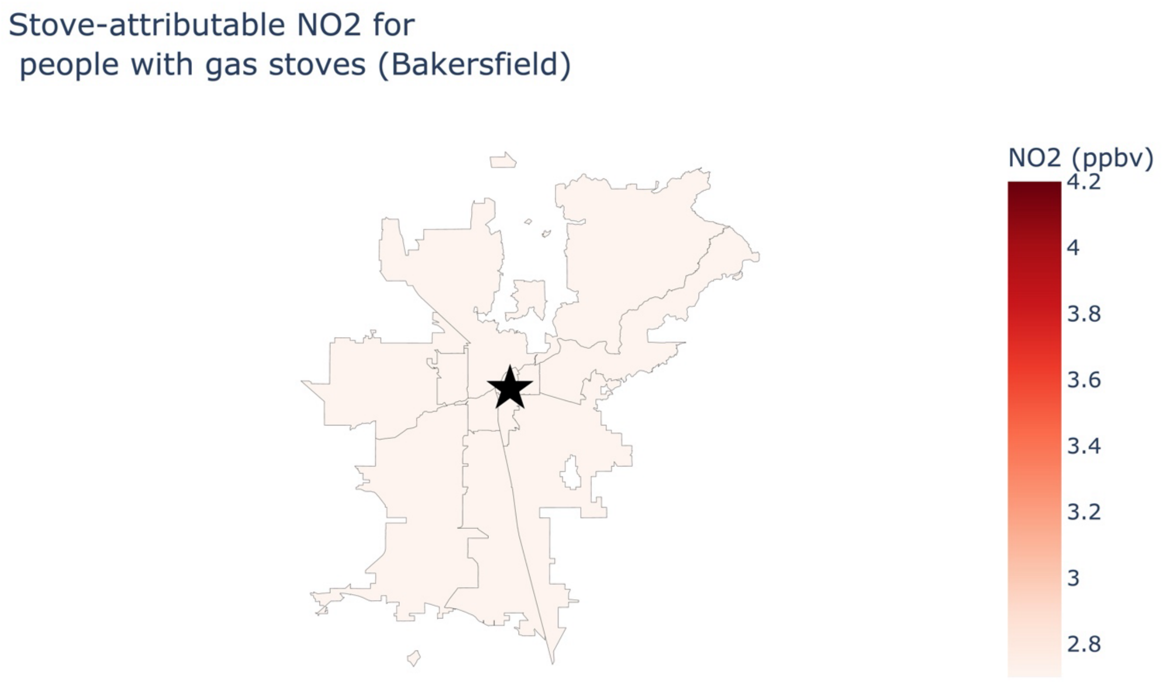

Fig. S11. Modeled stove-attributable long-term NO_2 exposure for people with gas or propane stoves in Bakersfield, CA. The black star indicates downtown.

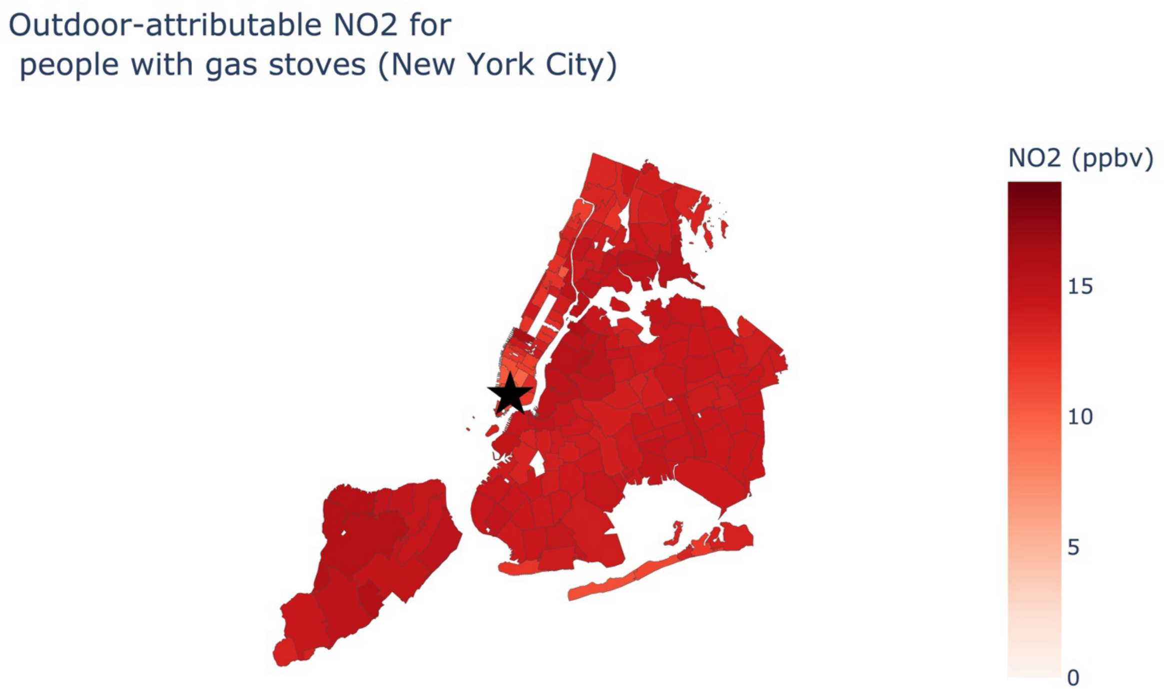

Fig. S12. Modeled outdoor-attributable long-term NO_2 exposure for people with gas or propane stoves in New York, NY. The black star indicates Lower Manhattan.

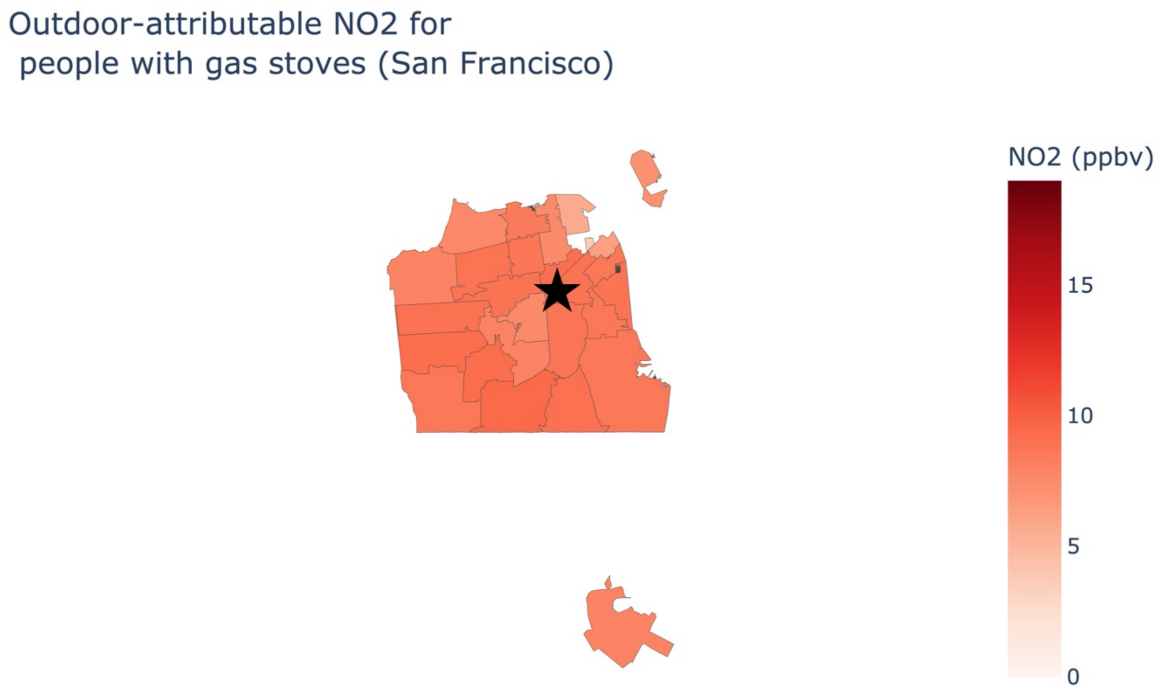

Fig. S13. Modeled outdoor-attributable long-term NO_2 exposure for people with gas or propane stoves in San Francisco, CA. The black star indicates the financial district.

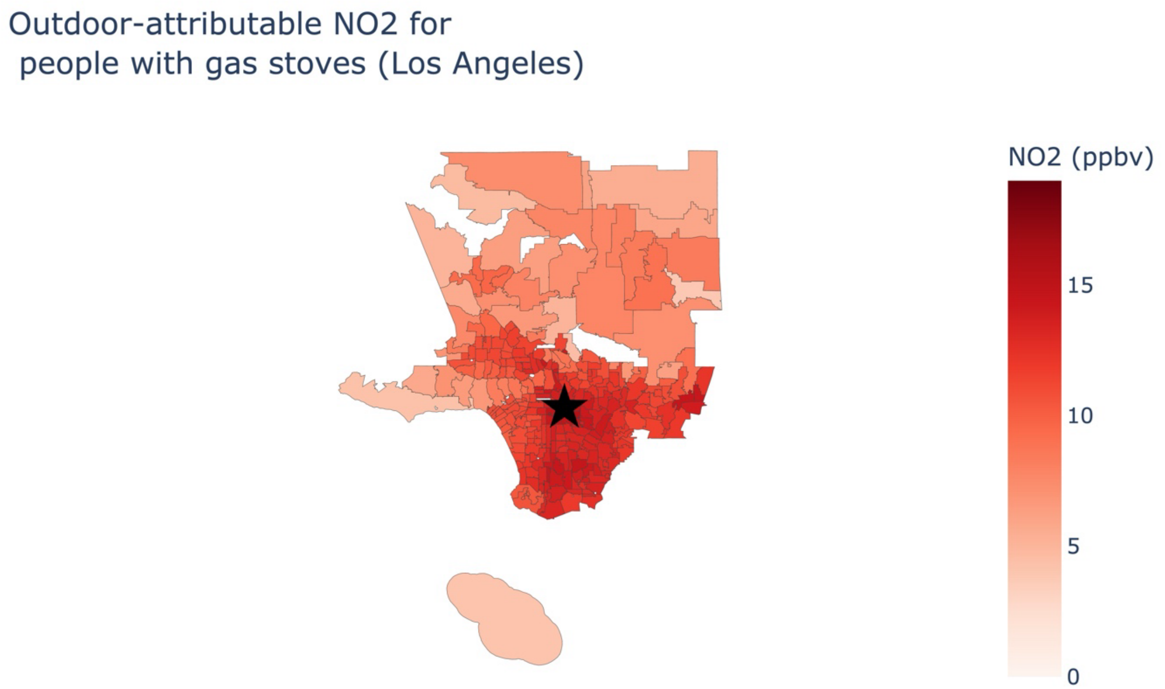

Fig. S14. Modeled outdoor-attributable long-term NO_2 exposure for people with gas or propane stoves in Los Angeles County, CA. The black star indicates downtown Los Angeles.

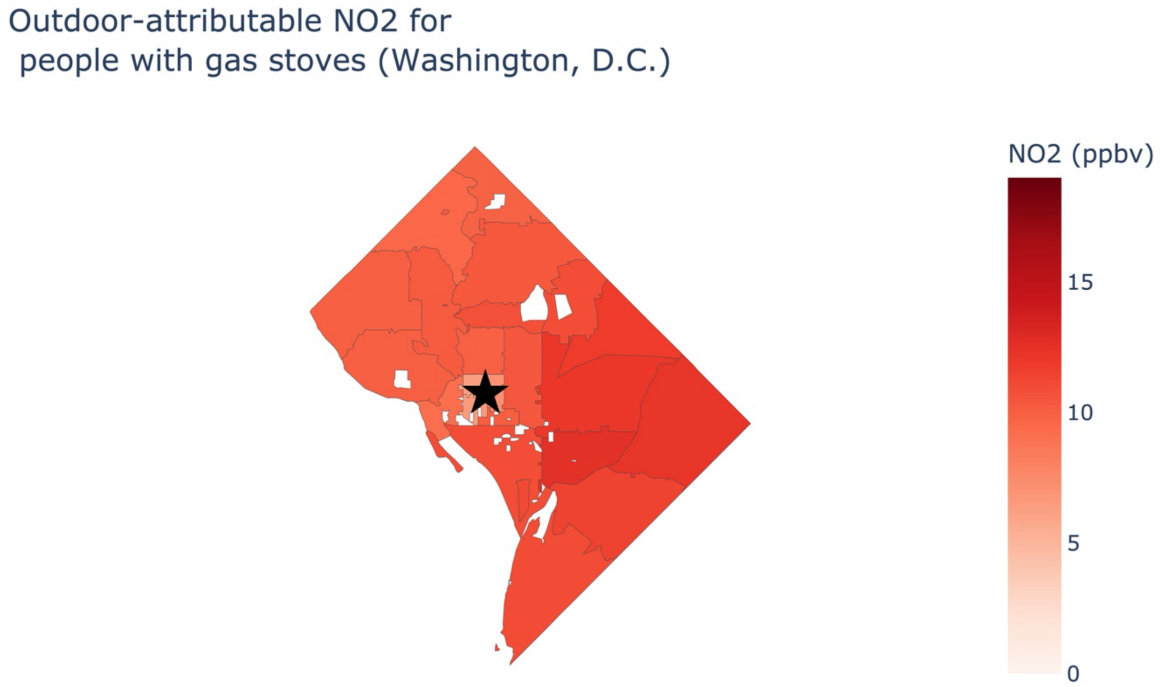

Fig. S15. Modeled outdoor-attributable long-term NO_2 exposure for people with gas or propane stoves in Washington, D.C. The black star indicates downtown.

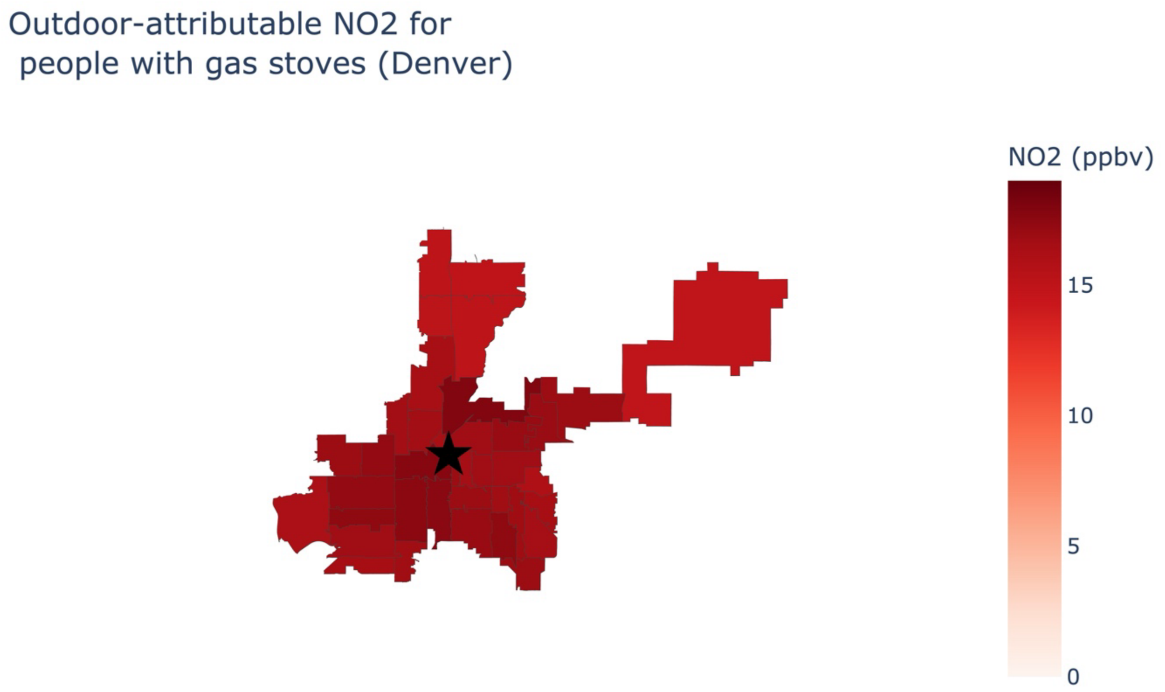

Fig. S16. Modeled outdoor-attributable long-term NO_2 exposure for people with gas or propane stoves in Denver, CO. The black star indicates downtown.

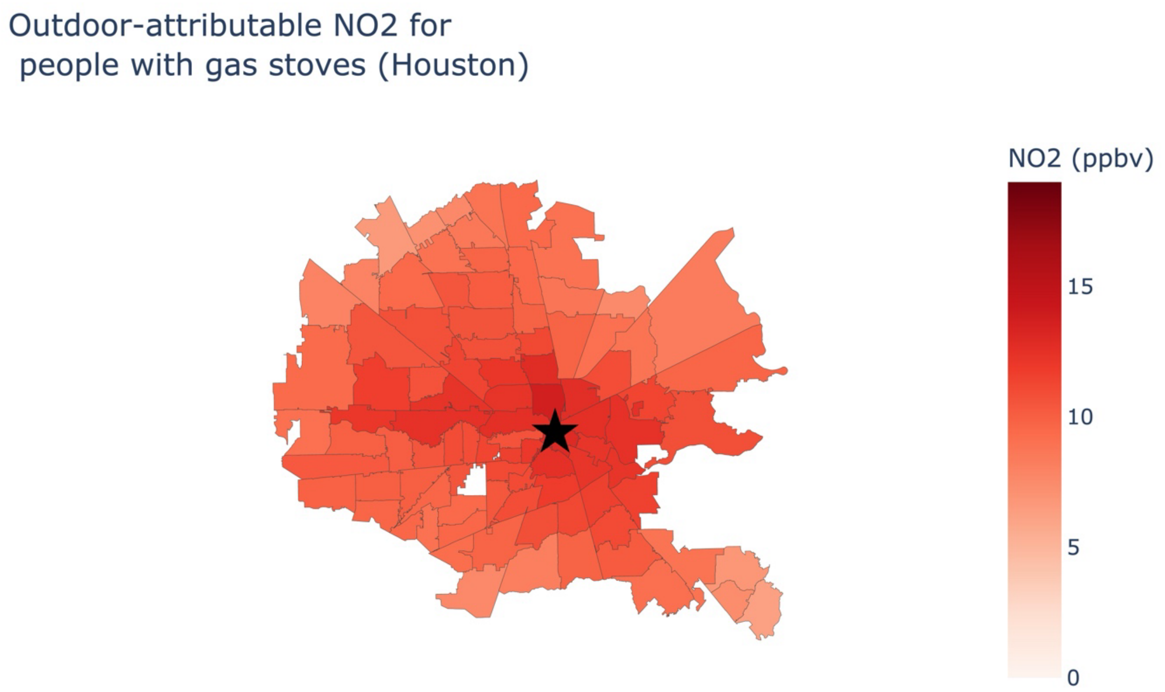

Fig. S17. Modeled outdoor-attributable long-term NO_2 exposure for people with gas or propane stoves in Houston, TX. The black star indicates downtown.

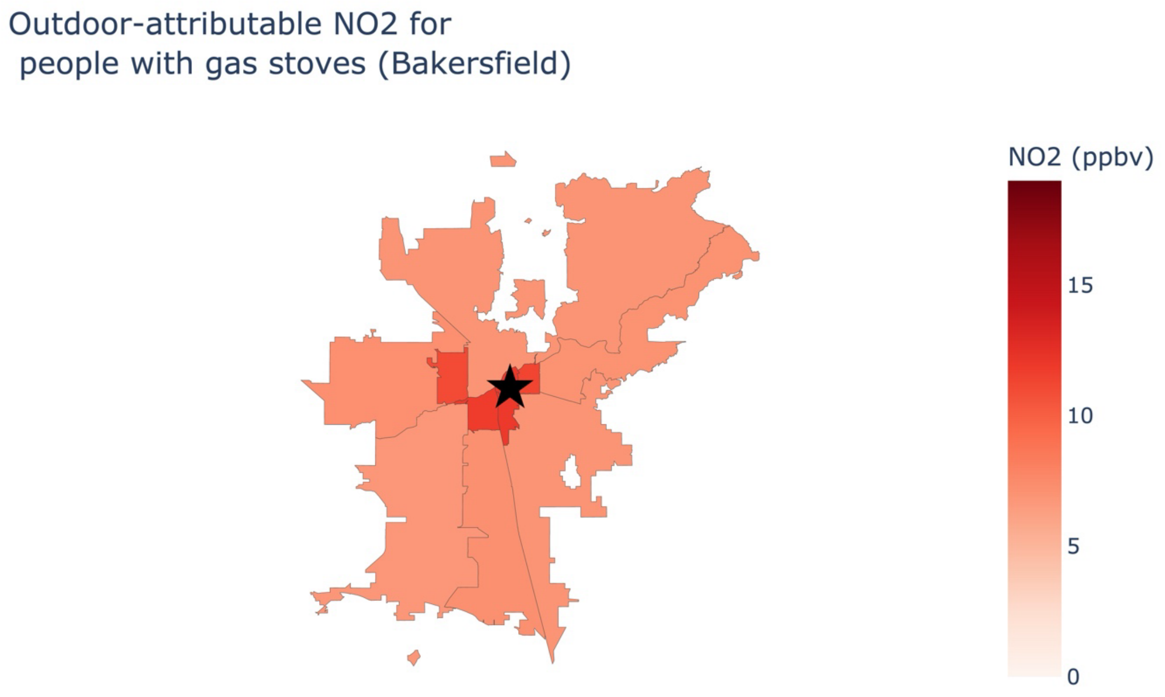

Fig. S18. Modeled outdoor-attributable long-term NO_2 exposure for people with gas or propane stoves in Bakersfield, CA. The black star indicates downtown.

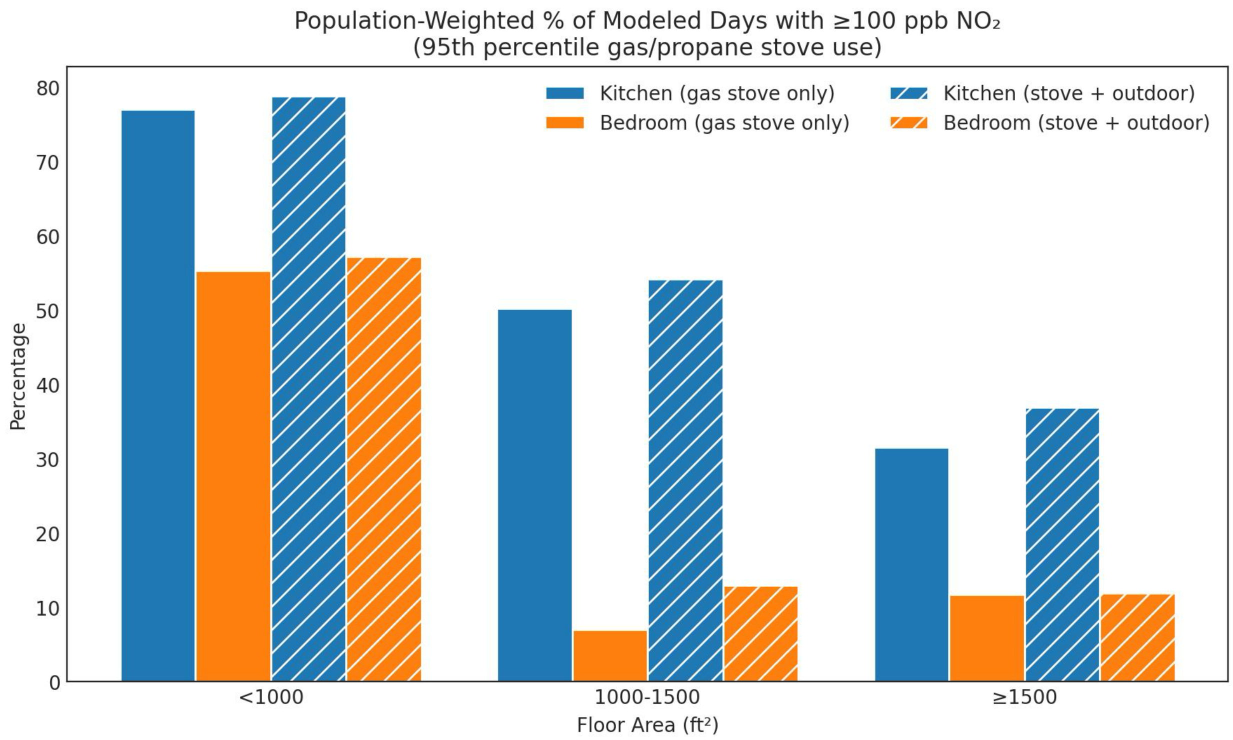

Fig. S19. Modeled percentage of days that NO_2 concentrations in the kitchen (blue) and bedroom (orange) exceed an hour-averaged value of 100 ppbv among households in the 95th percentile of gas or propane stove use. Solid colors represent exceedances due solely to gas or propane stove use while hatched bars represent exceedances due to the combination of outdoor-attributable NO_2 and NO_2 emitted by gas and propane stoves. Note that ≥1500 sq. ft. floorplans have more 100 ppbv exceedances in the bedroom attributable to gas and propane stoves only than do 1000-1500 sq. ft. floorplans. This is because the specifics of floorplan layout impact concentrations outside of the kitchen and aren't always fully offset by differences in total floorplan area.

**Hood Use and Capture Efficiency**		
Value	Weight	Sources and Explanation
No Hood or hood off during cooking or recirculating hood	79% for apartments; 74% otherwise	Hood use assumptions are based on a study (Zhao, H. et al., 2020) of 54 single-family homes and 17 low-income apartments in California in which hood use was monitored using anemometers (*2*). Zhao, H. et al. found that hoods are not used 72% of the time in apartments and 64% of the time in houses. Assumptions about the relative likelihood of different capture efficiencies being achieved are based on a study of 15 commonly-installed range hoods representing a spectrum of capture efficiencies (*7*). Assumptions about the prevalence of recirculating hoods (26% in homes with gas stoves) (*8*) and their inability to effectively remove NO_2 after > several weeks of use (*9*) from Sun and Wallace, 2021 and Jacobs and Cornelissen, 2017, respectively.
25% Capture efficiency outside-venting hood used while cooking	4%	
50% Capture efficiency outside-venting hood used while cooking	10% for apartments; 13% otherwise	
75% Capture efficiency outside-venting hood used while cooking	7% for apartments, 9% otherwise	

**Burner and Oven Use**			
Average daily burner-minutes and oven-minutes (mg NO_2 emitted and kJ of gas burned)	Scenario Used	Weight	Sources and Explanation
0.8 burner-minutes, 0 oven-minutes (0.64 mg NO_2 and 86 kJ of gas burned)	For instance: One day per week: One burner at 50% of high for 6 min in the evening. No oven used. Six days per week: no cooking.	10%	Assumptions about duration of cooking time are based on burner and oven usage measured directly using thermocouples in 54 single-family homes and 17 low-income apartments in California by Zhao et al., 2020 (*2*). The first, second, and third scenarios represent the 5th , 50th, and 95th percentile of total “burner-minutes” and “oven-minutes” measured. “Burner-minutes” are the number of burners on multiplied by minutes
39 burner-minutes, 0 oven-minutes (31 mg NO_2 and 4200 kJ of gas burned)	For instance: Two days per week: One burner at 50% of high for 5 min in the morning and two	90% if mixed fuel range (electric oven and gas cooktop),<br><br>80% otherwise.	

	burners at 50% of high for 22 min in the evening. No oven used.<br><br>Four days per week: Two burners at 50% of high for 22 min in the evening. No oven used.<br><br>One day per week: No cooking.		on (so one burner on for two minutes is the same as two burners on for one minute). Previous work has found that dinner contributes approximately 70% of total burner-minutes (*10*), in line with the scenarios presented here. The 5th percentile scenario was assigned to cooking from the 0th to 10th percentile (10% total), the 50th percentile scenario from the 10th to 90th percentile (80% total), the 95th percentile scenario from the 90th to the 100th percentile (10% total). Because acute (1-hour-averaged) exceedances may be biased high by this assignment, we also calculated exceedances with the 95th percentile weighted at 5% and reported these values in the results.<br><br>Since the median scenario does not use the oven, we weighted the median scenario in homes with mixed-fuel ranges at 90%.<br><br>We were unable to find quantitative data on the frequency of burner use at different intensities and thus relied on proxies. A 2011 survey of 372 California respondents (*10*) found that people boiled water (assumed to represent burners on high) in approx. 70% of meal preparations, pan- or stir-fried (assumed to represent burners on medium or medium-high) in approx. 60% of meals, and simmered (assumed to represent burners on low) in approx. 50% of meals. Based on this relatively even spread, assumed that "on" burners emit 25mL NO_2 h^{-1} (48 mg NO_2 h^{-1}), corresponding to half the emission
171 burner-minutes and 99 oven-minutes (199 mg NO_2 and 2700 kJ of gas burned)	For instance:<br>Seven days per week: Two burners at 50% of high for 13 min in the morning and at noon and two burners at 50% of high for 60 in the evening. Oven set to 350ºF for 99 min in the evening.	0% if mixed fuel range (electric oven and gas cooktop),<br><br>10% otherwise	

			rate of burners on high (Table 2) or just above medium heat (*11*).<br><br>We assumed that ovens were set to 350ºF and thus were on preheat for 9 minutes (*11*), emitting 70mL NO_2 h^{-1} (130 mg NO_2 h^{-1}), then emitting 10mL h^{-1} (18.5mg NO_2 h^{-1}) for the duration they were on. Values were proportionally scaled back from those reported in Lebel et al., since we modeled ovens to be on 350ºF only whereas Lebel et al. tested temperatures up to 425ºF.

Window Use

Value	Weight	Sources and Explanation
All windows closed all day	69% if ambient temp ≥ 15ºC<br>93% if ambient temp < 15ºC	Window opening assumptions are based on a survey by Sun and Wallace of the window-opening behavior of 132 individuals. The survey tracked how frequently respondents reported having kitchen windows open during cooking or immediately following cooking in the summer and winter (*8*).<br><br>To be conservative, we assumed that when windows were reported as open all windows added to the model floorplans were open (3 – 5 per floorplan, depending on number of bedrooms).
All modeled windows open for two hours at night, one hour in the morning, and one hour in the afternoon	30% if ambient temp ≥ 15ºC<br>7% fi ambient temp < 15ºC	
All modeled windows open all day	1% if ambient temp ≥ 15ºC<br>0% if ambient temp < 15ºC	

Ambient Temperature

Value	Explanation
0ºC	One cool season and one warm season temperature are calculated for each US Department of Energy Building Climate Zone (*12*), based on average winter and summer temperatures. “Subarctic” and “Very Cold” climates are assigned < 0ºC in the cool season and 10ºC in the warm season; “Cold” climates are assigned < 0ºC in the cool season and 20ºC in the warm season; “Mixed” and “Marine” climates are assigned 10ºC in the cool season and 30ºC in the warm season, and “Hot” climates are assigned 20ºC in the cool season and > 30ºC in the warm season.
10ºC	
20ºC	
30ºC	

Ambient Wind Speed

Value	Weights	Explanation
0	8.3%	Wind speed weights were calculated using the distribution of hour-averaged ground-level windspeeds measured between 2006 and 2020 at 1131 weather stations (ID numbers USW00003013 through USW00094996; see file wstation-wind.csv included in the downloadable data and code file) across the United States and reported by the National Centers for Environmental Information (*13*). Values less than 2.5 m/s were binned with 0, values between 2.5 m/s and 7.5 m/s were binned with 5 m/s, and values above 7.5 m/s were binned with 10 m/s.
5 m/s	60.8%	
10 m/s	30.8%	
**Occupancy**		
Value	Weights	Explanation
Default scenario	80%	Unless otherwise stated, we assume 35 minutes in the kitchen, 65 minutes outdoors, 9.5 hours in the bedroom and 6.5 hours neither outdoors (a period excluded from our estimate). These are the median values reported in the National Human Activity Pattern Survey (NHAPS) (*14*).<br><br>Except for the scenario with 5 minutes spent in the kitchen, we set occupancy schedules such that time spent in the kitchen was divided between the morning, beginning at 8:00am, and evening, beginning at 6:00pm. These coincided with burner and oven ignition times. For the "5 minutes in kitchen" scenario, time in the kitchen was spent entirely in the evening. See Supplementary Data S2 for tables containing each occupancy schedule.<br><br>Since we found that pollutant concentrations are not systematically different between non-kitchen rooms, we designated the remaining time as time spent in the living room.<br><br>We selected the remaining four scenarios based on the 5th and 95th percentiles of kitchen occupancy and time spent outside according to the NHAPS, leaving other variables unchanged. Occupancy is randomly assigned in the same manner as the other input variables and we do
5 minutes outside	5%	
5 minutes in the kitchen	5%	
8.5 hours outside	5%	
2.5 hours in the kitchen	5%	

		not assume that the occupancy schedule of one occupant is predictive of the schedule of other occupants.
**Forced Air System**		
We left forced air systems unchanged in all 24 floorplans. If a forced air system was present, we modeled it as being off for outdoor temperatures of 10ºC and 20ºC and on for outdoor temperatures of 0ºC and 30ºC.		

According to this model, chronic NO2 exposure is approximately 46% of the outdoor average concentration in a given ZIP code, though this value ranges from 38% to 49% in different ZIP codes and depends on housing stock and local climate. These infiltration values are in line with empirically-reported values in relatively small sets of test homes.

Table S1. Summary of the 24 floorplans selected from Persily et al. and used to model NO_2 exposure (*3*). "Living Space" excludes garages, external stairwells, unfurnished basements, and attics. These floorplans were previously used to estimate NO_2 exposure attributable to gas and propane stoves (*4*).

	Type	Living Space (sq. ft.)	Stories	Central Forced Air	Year Built
MH-1	Mobile Home	930	1	Yes	1970-1989
MH-2	Mobile Home	930	1	Yes	After 1990
MH-3	Mobile Home	930	1	No	1970-1989
MH-4	Mobile Home	930	1	Yes	1940-1969
DH-1	Detached Home	1880	1	Yes	1970-1989
DH-2	Detached Home	1140	2	Yes	1940-1969
DH-7	Detached Home	2080	2	Yes	1940-1969
DH-17	Detached Home	2020	3	Yes	1940-1969
DH-29	Detached Home	1152	1	No	Before 1940
DH-42	Detached Home	1030	1	No	1970-1989
DH-81	Detached Home	1740	2	No	Before 1940
APT-1	Apartment	700	1	Yes	1970-1989
APT-3	Apartment	690	1	No	1970-1989
APT-4	Apartment	700	1	No	Before 1940
APT-5	Apartment	700	1	No	1940-1969
APT-28	Apartment	1530	1	No	Before 1940
APT-35	Apartment	1400	1	Yes	After 1990
APT-62	Apartment	700	1	No	After 1990
AH-1	Attached Home	1040	3	Yes	Before 1940
AH-3	Attached Home	1040	1	Yes	1970-1989
AH-8	Attached Home	1040	1	No	1940-1969

AH-21	Attached Home	2030	2	Yes	Before 1940
AH-34	Attached Home	2840	3	Yes	1970-1989
AH-39	Attached Home	1040	1	Yes	After 1990

Table S2
Summary of model parameter values, relative weights, and explanation of value and weights choices, following Kashtan et al., 2024 (*4*).

Metro Area (including suburbs)	Stove-Attributable NO_2 Exposure Across the Population (ppbv)	Outdoor-Attributable NO_2 Exposure (ppbv)
New York, NY	3.5	14
San Francisco, CA	3.4	8.6
Washington, D.C.	3.0	11
Los Angeles, CA	2.8	12
Denver, CO	2.7	17
Santa Cruz, CA	2.7	3.2
Houston, TX	2.4	10
Bakersfield, CA	2.3	8.7

Table S3. Population-weighted mean long-term stove-attributable NO_2 exposure across the population with gas or propane stoves and long-term outdoor-attributable NO_2 exposure.

Supplementary Data S1. Table reporting exposure values by city (leftmost column). The values are, from left to right: long-term stove-attributable NO_2 exposure for people with gas and propane stoves, outdoor-attributable exposure, total long-term NO_2 exposure for people with gas and propane stoves, and total long-term NO_2 exposure across the entire population including people with electric stoves.

Supplementary Data S2. Folder containing five tables reporting our modeled occupancy schedules (see Table S2). Times are listed in hours starting at 7am of the model day, such that 0 corresponds with 7am. The files are as follows: median.csv corresponds with the default scenario, assigned a weight of 0.8. fifth_kitchen.csv and ninetyfifth_kitchen.csv correspond with the 5th and 95th percentiles of time spent in the kitchen, respectively. fifth_outside.csv and ninetyfifth_outside.csv correspond with the 5th and 95th percentiles of time spent outdoors, respectively.

References

1. US Census Bureau, ZIP Code Tabulation Areas (ZCTAs). https://www.census.gov/programs-surveys/geography/guidance/geo-areas/zctas.html.

2. H. Zhao, W. R. Chan, W. W. Delp, H. Tang, I. S. Walker, B. C. Singer, Factors Impacting Range Hood Use in California Houses and Low-Income Apartments. *Int. J. Environ. Res. Public Health 2020 Vol 17 Page 8870* **17**, 8870 (2020).

3. A. K. Persily, A. Musser, D. D. Leber, A Collection of Homes to Represent the U.S. Housing Stock (2008). https://www.nist.gov/publications/collection-homes-represent-us-housing-stock.

4. Y. Kashtan, M. Nicholson, C. J. Finnegan, Z. Ouyang, A. Garg, E. D. Lebel, S. T. Rowland, D. R. Michanowicz, J. Herrera, K. C. Nadeau, R. B. Jackson, Nitrogen dioxide exposure, health outcomes, and associated demographic disparities due to gas and propane combustion by U.S. stoves. *Sci. Adv.* **10**, 8680 (2024).

5. Download Files | AirData | US EPA. https://aqs.epa.gov/aqsweb/airdata/download_files.html#Raw.

6. Downloads » Daily and Annual PM2.5, O3, and NO2 Concentrations at ZIP Codes for the Contiguous U.S., v1: Air Quality Data for Health-Related Applications | SEDAC. https://sedac.ciesin.columbia.edu/data/set/aqdh-pm2-5-o3-no2-concentrations-zipcode-contiguous-us-2000-2016/data-download.

7. B. C. Singer, W. W. Delp, P. N. Price, M. G. Apte, Performance of installed cooking exhaust devices. *Indoor Air* **22**, 224–234 (2012).

8. L. Sun, L. A. Wallace, Residential cooking and use of kitchen ventilation: The impact on exposure. *J. Air Waste Manag. Assoc.* **71**, 830–843 (2021).

9. P. Jacobs, E. Cornelissen, Efficiency of recirculation hoods with regard to PM2.5 and NO2.

10. V. Klug, A. Lobscheid, B. Singer, "Cooking Appliance Use in California Homes" (Lawrence Berkeley National Laboratory (LBNL), Berkeley, CA (United States), 2011); https://doi.org/10.2172/1050839.

11. E. D. Lebel, C. J. Finnegan, Z. Ouyang, R. B. Jackson, Methane and NOx Emissions from Natural Gas Stoves, Cooktops, and Ovens in Residential Homes. *Environ. Sci. Technol.* **56**, 2529–2539 (2022).

12. Climate Zones | Department of Energy. https://www.energy.gov/eere/buildings/climate-zones.

13. Hourly Ground-Level Wind Data | National Centers for Environmental Information (NCEI). https://www.ncei.noaa.gov/access/search/data-search/normals-hourly-2006-2020?dataTypes=HLY-WIND-AVGSPD.

14. N. E. Klepeis, A. M. Tsang, J. V. Behar, “Analysis of the National Human Activity Pattern Survey (NHAPS) Respondents from a Standpoint of Exposure Assessment” (1995).
